# Metabolomic and Transcriptomic Profiles in Diverse *Brassica oleracea* Crops Provide Insights into the Genetic Regulation of Glucosinolate Profiles

**DOI:** 10.1021/acs.jafc.4c02932

**Published:** 2024-07-08

**Authors:** Chengcheng Cai, Ric C.H. de Vos, Hao Qian, Johan Bucher, Guusje Bonnema

**Affiliations:** † Plant Breeding, Wageningen University and Research, Wageningen 6708 PB, The Netherlands; ‡ State Key Laboratory of Vegetable Biobreeding, Key Laboratory of Biology and Genetic Improvement of Horticultural Crops of the Ministry of Agriculture and Rural Affairs, Sino-Dutch Joint Laboratory of Horticultural Genomics, Institute of Vegetables and Flowers, Chinese Academy of Agricultural Sciences, Beijing 100081, China; § Bioscience, Wageningen University and Research, Wageningen 6708 PB, The Netherlands

**Keywords:** *Brassica oleracea*, glucosinolates, transcriptome, secondary metabolites

## Abstract

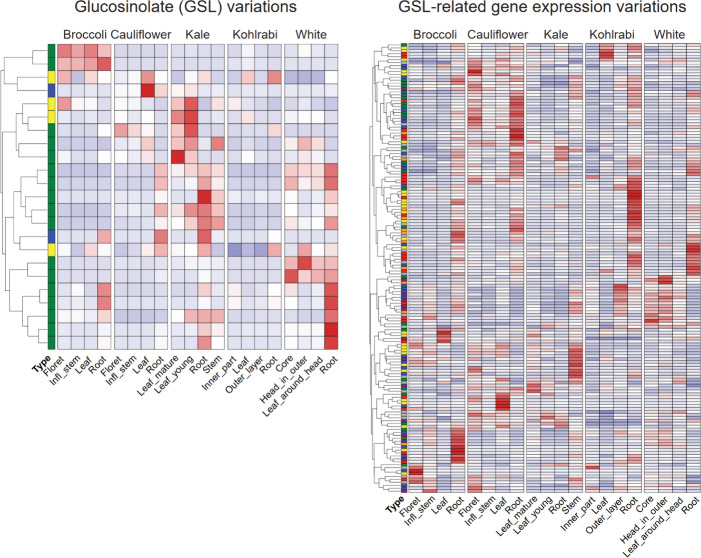

Glucosinolates (GSLs) are plant secondary metabolites commonly found in the cruciferous vegetables of the Brassicaceae family, offering health benefits to humans and defense against pathogens and pests to plants. In this study, we investigated 23 GSL compounds’ relative abundance in four tissues of five different *Brassica oleracea* morphotypes. Using the five corresponding high-quality *B. oleracea* genome assemblies, we identified 183 GSL-related genes and analyzed their expression with mRNA-Seq data. GSL abundance and composition varied strongly, among both tissues and morphotypes, accompanied by different gene expression patterns. Interestingly, broccoli exhibited a nonfunctional *AOP2* gene due to a conserved 2OG-FeII_Oxy domain loss, explaining the unique accumulation of two health-promoting GSLs. Additionally, transposable element (TE) insertions were found to affect the gene structure of *MAM3* genes. Our findings deepen the understanding of GSL variation and genetic regulation in *B. oleracea* morphotypes, providing valuable insights for breeding with tailored GSL profiles in these crops.

## 1 Introduction

Glucosinolates (GSLs) are a group of secondary plant metabolites almost exclusively found in the order Brassicales*.*^1^ These GSL compounds, derived from glucose and amino acids, are rich in nitrogen and sulfur and are water-soluble.^2,3^ GSLs share a common core structure, which is linked to an amino acid derived side-chain, with thioglucose and sulfate groups.^4^ According to their precursor amino acid, GSLs can be classified as aliphatic GSLs (derived from alanine, isoleucine, leucine, methionine, and valine), aromatic GSLs (derived from phenylalanine and tyrosine), or indolic GSLs (derived from tryptophan).^1,3^ Briefly, the biosynthesis of aliphatic and aromatic GSLs includes three independent stages: side-chain elongation of the precursor amino acid, formation of the core structure, and side-chain modification, whereas indolic GSL biosynthesis includes only core structure formation and side-chain modification processes.^5^ GSLs are extremely variable due to the differences in side-chains, chain lengths, and additional side-chain modifications. To date, more than 130 GSL structures are scientifically documented in *Arabisopsis thaliana* and other plants.^6^ In plants, the hydrolysis products of GSLs play important roles in defense against pathogens and pests.^7^ In vegetables, they provide diverse tastes like bitterness and pungency.^8^ In addition, GSLs have been reported to have both antinutritional and health-promoting effects.^3^ For example, increasing evidence points to a cancer prevention and anti-inflammatory effect of isothiocyanates (ITCs) that are produced from GSLs upon cell damage.^9^ However, some GSLs are antinutritional, such as progoitrin that promotes goiter disease.^10^

*Brassica oleracea* is an economically important vegetable and fodder crop species cultivated worldwide. It consists of many morphotypes that exhibit an enormous diversity in their appearance. For example, cabbages (var. *capitata*) form leafy heads, with different varieties differing in leaf color and texture and/or head shape; broccoli (var. *italica*) and cauliflower (var. *botrytis*) are characterized by their typical curd with large arrested inflorescences; kohlrabi (var. *gongylodes*) forms enlarged tuberous stems; kales (var. *acephala*) are characterized by their variation in leaf shapes, color, and structure, including bore and curly kale, marrow stem kale, etc.^11,12^ Despite this enormous diversity, *B. oleracea* truly is a single species, and morphotypes can be easily interbred. Several studies clearly showed that the genomes of the different morphotypes contain numerous structural variations and that their gene contents can vary extensively.^13,14^ Besides diversity in appearance and genome sequence, *B. oleracea* crops also vary remarkably in their GSL content and composition. For example, Yi et al.^15^ determined the content of 16 different types of GSLs in edible organs of 12 *B. oleracea* genotypes, including four different morphotypes. Hahn et al.^16^ estimated the content of 5 GSLs in 25 kale varieties and 11 nonkale *B. oleracea* cultivars. Similarly, Bhandari et al.^17^ assessed the content of 12 types of GSLs in the head of 146 cabbage genotypes. All these studies showed that GSL composition and levels differed markedly among different *B. oleracea* genotypes.

To date, most of the knowledge with regard to biosynthesis, degradation, transport, and regulation mechanisms as well as the function of GSLs is based on extensive studies performed in *A. thaliana*, including mutant screens, quantitative trait loci (QTL) mapping, and genome-wide association studies (GWAS).^5,18^ Indeed, a total of 113 genes controlling GSL biosynthesis, degradation, transport, and storage have been experimentally characterized in this model species, including 85 enzyme-encoding genes, 23 transcription factors, and 5 transporter proteins.^6^ Comparative genomic analyses between *Arabidopsis* and other *Brassica* crops can provide comprehensive information for the GSL biosynthetic pathway in these nonmodel crops.^15,19^ Recently, we *de novo* assembled five high-quality chromosome-scale *B. oleracea* genomes,^13^ which provide valuable genomic resources for identifying GSL related genes in *B. oleracea* and studying their sequence divergence. From the aspect of breeding, an important goal is to generate optimal GSL profiles with regard to health and taste in the edible organs of *B. oleracea* and retain the plant growth protective effects such as the pest insect damage protection and the inhibition of weed growth in surrounding areas.^20^ To do so, it is key to better understand the genetic regulation of GSL variation between genotypes and tissues in *B. oleracea*.

Here, we analyzed the relative abundance of 23 different GSL structures in four different plant tissues, including roots, stems, and the edible parts of five different *B. oleracea* morphotypes. Based on all the experimentally characterized GSL genes in *Arabidopsis*, we identified their homologues in the corresponding five high-quality *B. oleracea* genome assemblies. Moreover, mRNA-Seq was generated for the same samples that were used for GSL profiling to study gene expression. We revealed strong variation in terms of composition and relative abundance of GSLs among different tissues and different morphotypes. The identified GSL-related genes were differently expressed between different tissues and different morphotypes. We found significant correlations between the abundance of 23 GSLs and expression level of 109 related genes. We also present interesting observations in this study, including a nonfunctional *AOP2* in broccoli related to the loss of the conserved 2OG-FeII_Oxy domain, explaining the specific accumulation of desired 4-methylsulfinylbutyl and 5-methylsulfinylpentyl GSLs in broccoli, and transposable element (TE) insertion activities in one paralogue of the *MAM3* gene in three out of the five genomes causing long and repeat-rich introns, respectively.

## 2 Materials and Methods

### 2.1 Plant Materials and Sample Collection

For the current study, we used five homozygous genotypes (broccoli, cauliflower, kale, kohlrabi, and white cabbage) for metabolite extraction and mRNA sequencing, the genomes of which were previously *de novo* assembled.^13^ The seeds were sown in April 2020 in a single greenhouse compartment at Unifarm (Wageningen University and Research), and samples were harvested between 65 and 143 days after sowing (Figure S1). A completely randomized block design with three blocks was used for plant growth. Over each block, three plants of each accession were randomly distributed. As shown in Figure S1, we collected four different tissues from each accession with three biological replicates. For each biological replicate, equal weight of tissue from the three plants per accession in the same block was pooled. These samples were immediately frozen in liquid nitrogen, ground into a fine powder, and stored at −80 °C until further use.

### 2.2 Glucosinolate Profiling

Intact GSLs were determined using HPLC coupled to accurate mass detection (LCMS). We previously showed a good correlation between data obtained from the analyses of GLSs as their intact sulfate forms using LCMS and as their desulfated forms using conventional HPLC with UV detection.^21^ However, the HPLC-UV protocol is more laborious because of the essential desulfatase treatment and extract cleanup, and UV detection is also less specific and less sensitive as compared to accurate MS. In the present study, the intact GSLs were extracted from 200 mg powder to which 600 μL of 99.87% methanol containing 0.13% formic acid was added followed by 15 min sonication and then 15 min centrifugation at 16,000*g*. The clear supernatants were directly used for LCMS analysis conforming with Jeon et al.,^22^ using a Dionex U-HPLC coupled to a Q-Exactive Orbitrap FTMS (Thermo Scientific, USA). In short, 5 μL of each extract was injected, and compounds were separated on a C18 column (Phenomenex Luna, 2.0 × 150 mm, 3 μm particle size) using a 45 min gradient from 5 to 75% acetonitrile in MQ acidified with 0.1% formic acid. Electrospray ionization in negative mode in the *m*/*z* range of 90–1350 at a mass resolution of 60,000 fwhm was used to detect eluting compounds. The spray voltage was 3500, capillary temperature 290 °C, sheath gas 40, auxiliary gas 10, spare gas 1.9, probe heater 60 °C, and S-lens RF level 50. GSLs were identified based on the relative retention times and observed accurate masses,^2^ allowing a maximum deviation of 3 ppm from the calculated molecular ion [M – H]^−^. Chromatographic peak areas of GSLs were subsequently integrated using the QualBrowser module of Xcalibur version 4.1 (Thermo Scientific, USA). Because plant extracts were all similarly and simultaneously prepared with the same ratio of extraction solvent versus sample weight, for each individual GSL, the obtained relative abundance values (LCMS peak areas) can be directly compared across samples.

### 2.3 mRNA Extraction and Sequencing

Total RNA was extracted from the frozen powders using the TRIZOL reagent (Invitrogen, Carisbad, CA, USA) according to the manufacturer’s protocol and treated with RNase-free DNase I (Invitrogen, Carisbad, CA, USA) to remove genomic DNA contaminations. Total RNA was cleaned using the cleanup protocol of the RNeasy Mini Kit (Qiagen, The Netherlands) according to the supplier’s recommendations. RNA quantity and quality were assessed using a NanoDrop One Spectrophotometer (Thermo Fischer Scientific, USA), agarose gel electrophoresis, and a Qubit RNA BR Assay Kit (Thermo Fisher Scientific, USA) on a Qubit 4 Fluorometer. mRNA-Seq libraries were prepared using the Illumina TruSeq RNA Sample Prep Kit and sequenced on the Illumina NovaSeq platform with 150 bp paired-end reads.

### 2.4 Read Mapping and Gene Expression Profiling

Low-quality reads were removed using fastp (v0.19.5)^23^ with parameters “-q 15 -u 40 -n 5 -l 100 --trim_poly_x --detect_adapter_for_pe”. To minimize alignment errors, we mapped the clean reads to the five corresponding reference genomes^13^ using Hisat2 (v2.1.0)^24^ with parameters “--dta”. Read counts for each gene were computed using htseq-count (part of the HTSeq version 0.12.4)^25^ with parameters “-s no -q -f bam -r pos”. Hierarchical clustering and principal component analysis (PCA) were performed using the PCAPlot and clusterPlot function in the SARTools (v1.8.1) package^26^ to check qualities for mRNA-seq replicates. Stringtie (v2.1.1)^27^ was utilized to compute the expression level of genes in terms of transcripts per kilobase of exon model per million mapped reads (TPM), with parameters “-e -B”. Genes with an average TPM ≥ 1 across the three biological replicates were considered as expressed. If a gene is expressed in any tissue of a morphotype, it is considered as expressed for the given morphotype.

### 2.5 Identification of GSL Genes in Five *B. oleracea* Morphotypes

OrthoFinder (v2.3.12)^28^ was used to detect orthologs based on protein sequences from five *B. oleracea*(13) and one *A. thaliana* genomes (TAIR10) with default parameters. A comprehensive *A. thaliana* GSL gene list was obtained from Harun et al.,^6^ which includes a total of 113 genes encoding 85 enzymes, 23 transcriptional components, and 5 protein transporters. Sequences of these GSL genes were retrieved from the TAIR database (https://www.arabidopsis.org/). To identify GSL genes in the five *B. oleracea* genomes, all *A. thaliana* GSL genes were compared with the orthologs identified between *A. thaliana* and each of the five *B. oleracea* genomes. We extracted all the *B. oleracea* genes that are orthologous to *A. thaliana* GSL genes. Subsequently, we filtered these candidate GSL homologues based on blastp alignments between all *B. oleracea* and *A. thaliana* GSL protein sequences with a cutoff *E* value ≤ 1 × 10^–20^, coverage ≥ 50%, and identity ≥ 35%.

### 2.6 Motif and Domain Analysis and Multiple Sequence Alignment

The MEME (https://meme-suite.org/meme/) tool was used to identify conserved motifs for selected GSL genes in *B. oleracea* genomes, with a maximum number of 10 motifs and a motif width of 6–50.^29^ NCBI-CDD (https://www.ncbi.nlm.nih.gov/Structure/bwrpsb/bwrpsb.cgi) was used to search for the conserved domains. The CFVisual software (https://github.com/ChenHuilong1223/CFVisual) was used to visualize the gene structure and distribution of motifs and domains.^30^ Protein sequences of interested genes were aligned using MAFFT^31^ with default parameters and were then visualized with Jalview 2 (v2.11.2.4).^32^

### 2.7 Statistical Analysis

Morphotype signature GSLs were identified by comparing the average relative GSL levels across the four tissues among the five morphotypes using the Student–Newman–Keuls test with a significance level of α = 0.05. The correlation between the accumulation of GSLs and the expression of GSL-related genes in *B. oleracea* was analyzed by calculating Pearson’s correlation coefficient using *R*. A *p* value of less than 0.05 was considered a significant correlation.

## 3 Results

### 3.1 GSL Profile Variations among *B. oleracea* Morphotypes and Tissues

Intact GSLs were analyzed in five *B. oleracea* morphotypes (broccoli, cauliflower, kale, kohlrabi, and white cabbage), with each morphotype including four different tissues from plants grown in three biological replicates (Figure S1). We identified a total of 23 different GSLs using LCMS, consisting of 17 aliphatic, 2 aromatic, and 4 indolic GSLs (Table S9). Analytical variation was determined for each GSL by analyzing so-called quality control samples (QCs), which consisted of five independent extractions from a large pooled sample prepared by mixing the same small amount of each biological sample. These QCs were similarly and jointly prepared with the biological samples. One QC was analyzed before and one QC after the entire series, and the remaining three QCs were evenly distributed between the 60 real samples. Based on the chromatographic GSL peak areas obtained with these five QCs (Table S9), the analytical variation of the GSLs in the *B. oleracea* samples was on average 11.2%, ranging from 1.1% for 4-hydroxy-3-indolylmethyl-GSL to 28.9% for 8-methylsulfinyloctyl-GSL.

**Figure 1:**
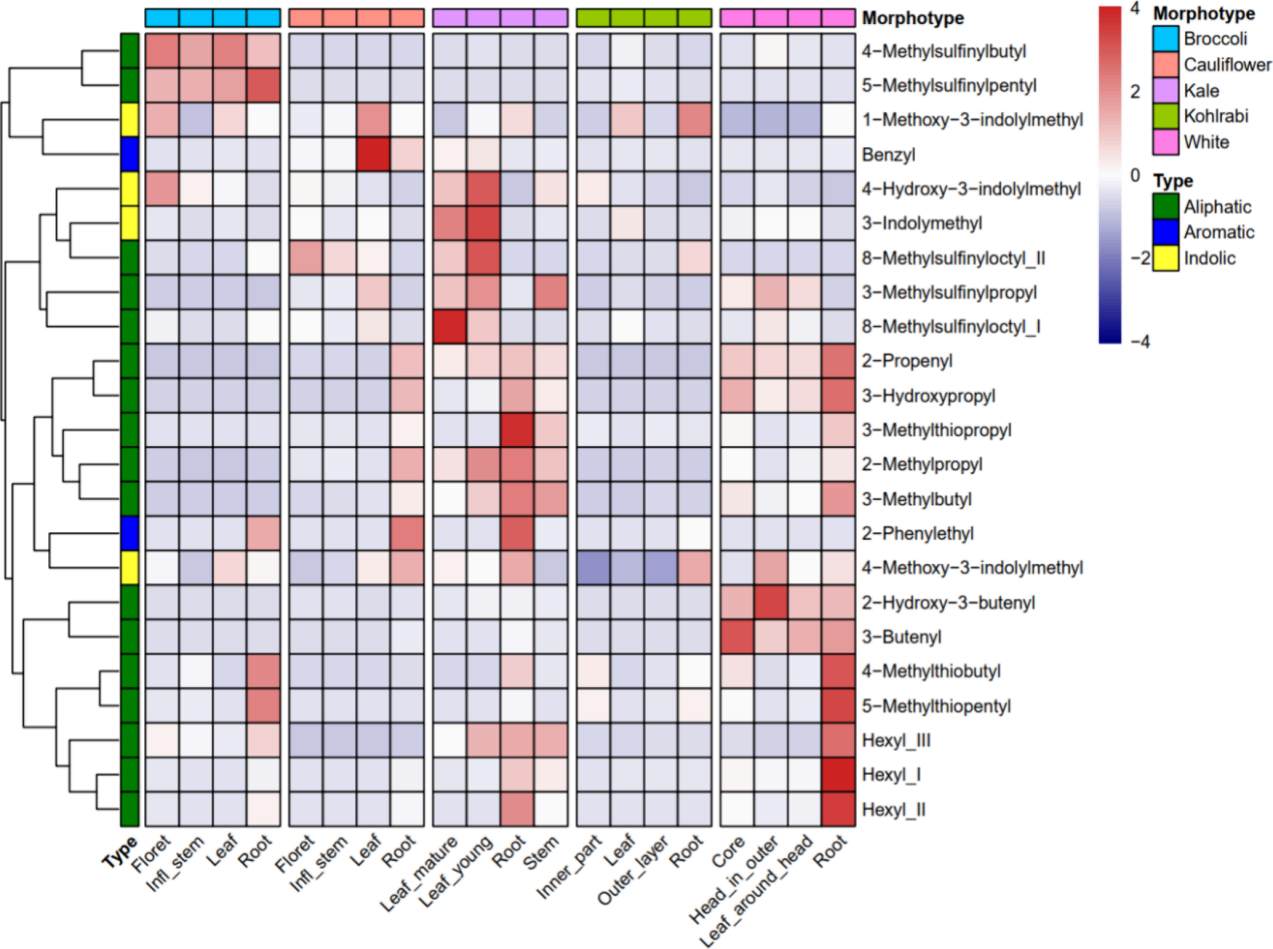
Variations in glucosinolate (GSL) levels. For each tissue, the average relative intensity value of the three biological replicate samples was considered as the GSL concentration (note: each sample consists of 200 mg FW extracted with 600 μL solvent). Per GSL, the relative intensity values were normalized across samples using *Z*-score standardization.

Principal component analysis (PCA) based on GSL data showed close positions between the three biological replicates for the vast majority of sampled tissues, indicating relatively low biological variations (Figure S2). We found extensive variation in GSL profiles among different morphotypes as well as between different tissues by comparing the relative abundances of each compound (Figure 1). Overall, kohlrabi showed relatively low abundance of nearly all detected GSLs, whereas kale and white cabbage exhibited relative high levels of most GSLs. We detected morphotype signature GSLs of which the average abundance across the four tissues in one morphotype was significantly higher than that in any other morphotype (Student–Newman–Keuls test with α = 0.05). Accordingly, kale and white cabbage contain nine and eight signature GSLs, respectively (Table S1). In contrast, we did not find any kohlrabi signature GSL among these 23 compounds. Three compounds (1-methoxy-3-indolylmethyl, 8-methylsulfinyloctyl_II, and 4-methoxy-3-indolylmethyl) were not signature for any morphotype. Two broccoli signature GSLs (4-methylsulfinylbutyl and 5-methylsulfinylpentyl) showed relatively high levels in all its tissues, whereas their levels were remarkably lower in any tissue of the other morphotypes. This was also the case for two of the white cabbage signature GSLs (3-butenyl and 2-hydroxy-3-butenyl) (Figure 1 and Figure S3). In the profiled 23 GSLs, we observed that broccoli lacked C3 aliphatic GSLs (Figure S3a). Similarly, we found either remarkably low abundance or no accumulation of C3 aliphatic GSLs in kohlrabi tissues (Figure S3a). Cauliflower did not accumulate C4 and C5 aliphatic GSLs in any tissue (Figure S3b,c). Generally, most detected GSLs tend to be accumulated at a higher level in roots than in other tissues (Figure 1 and Figure S3). For example, the aromatic 2-phenylethyl GSL was detected at relatively high levels only in roots of the three morphotypes: broccoli, cauliflower, and kale.

### 3.2 GSL-Related Gene Identification in *B. oleracea* Genomes and Their Overall Expression

In *Arabidopsis*, a total of 113 GSL-related genes have been identified, including 85 GSL biosynthesis genes, 23 transcriptional components, and 5 transporters.^6^ Using these genes as queries, we identified a total of 183 nonredundant orthologous genes in the five high-quality *B. oleracea* genome assemblies,^13^ including 167, 165, 170, 166, and 170 genes in broccoli, cauliflower, kale, kohlrabi, and white cabbage, respectively, which are distributed all along the nine *B. oleracea* chromosomes (Tables S2 and S3 and Figure S4). The vast majority (153 genes) of these genes showed one-to-one syntenic relationship among the five genomes (Table S2). However, we also found a total of 30 presence and absence genes among the five genomes. Across the 113 GSL-related genes in *Arabidopsis*, we observed that 50 of them had multiple orthologous gene copies (≥2) in at least one of the five *B. oleracea* genomes, with the copy number differing among the five morphotypes for several genes. As an example, one and four homologous copies of *MYB51* were found in broccoli and kale, respectively. Also, 42 out of the 113 *Arabidopsis* GSL genes had a maximum of one copy in each of the five *B. oleracea* genomes. However, for some genes, such as *NSPs*, *FMO*_*GS-OXs*_, and *NITs*, less paralogous copy numbers were found in *B. oleracea* than in *Arabidopsis*. Additionally, two *Arabidopsis* genes (*CCA1* and *MYB115*) had no orthologues in any of the five *B. oleracea* genomes. The expanded number of GSL related genes in *B. oleracea* is partly attributed to the whole genome triplication (WGT) event.^33,34^ However, because of the extensive gene fractionation that occurred following the WGT,^33,34^ less than three copies and even no orthologues in *B. oleracea* were also found for some GSL homologues (Table S2).

Paired-end mRNA-seq was performed for the same samples that were used for GSL profiling. We generated a total of 3.08 × 10^9^ clean reads (∼460.53 Gb) from the 60 samples (20 tissues × 3 biological repeats), averaging 5.13 × 10^7^ clean reads (7.68 Gb) per sample (Table S4). On average, 91.33% of these clean reads were concordantly and uniquely mapped to the five corresponding reference genomes. Quality control by PCA and hierarchical clustering analysis showed that the three biological replicates closely clustered together, indicating the good repeatability of gene expression data between biological replicates (Figure S5). Different tissues of each morphotype were clearly separated by PC1, PC2, or PC3. Accordingly, the hierarchical clustering analysis divided the samples of each morphotype into four groups, representing four different tissues (Figure S5). Generally, the roots are the most derived tissues that are separated from the remaining tissues. We estimated gene expression levels by transcripts per million (TPM) based on the alignments of mRNA-Seq reads.

**Figure 2:**
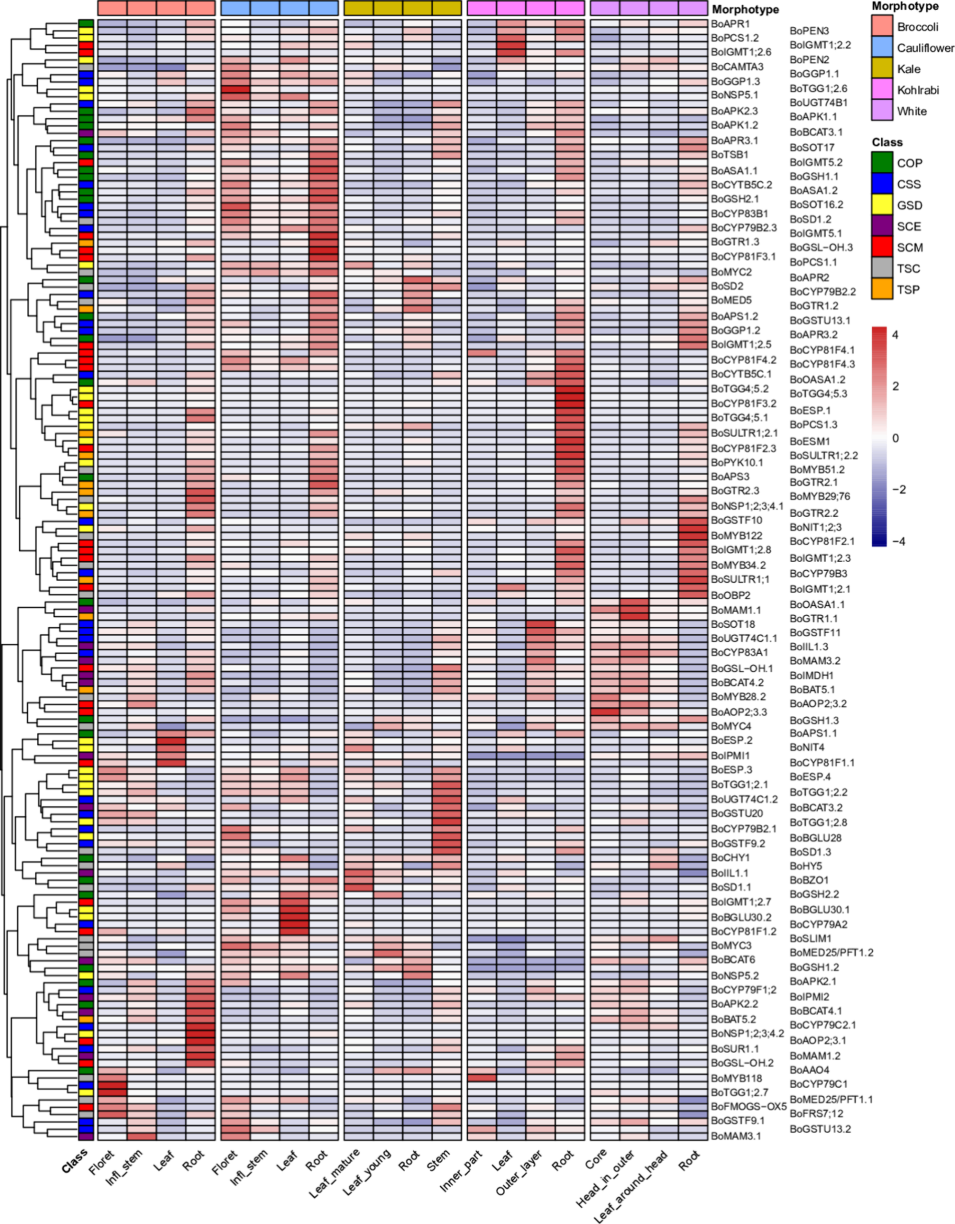
Expression profiles for GSL-related genes in four tissues of five *B. oleracea* morphotypes. Heatmaps were constructed using *z*-scored TPM values. Blue and red colors are used to represent low and high expression levels, respectively. Genes are classified based on their involvement in different processes/phases (abbreviations: COP: cosubstrate pathways, CSS: core structure synthesis, GSD: GSL degradation, SCE: side-chain elongation, SCM: side-chain modification, TSC: transcriptional components, and TSP: transporters).

We then investigated the overall expression pattern of the 153 one-to-one syntenic GSL genes among different *B. oleracea* morphotypes and tissues by constructing heatmaps combined with a hierarchical clustering analysis based on gene expression profiles (log2 transformed and *z*-scored TPM values, separately). Three clusters were revealed based on the log2 transformed TPM values, with genes in cluster I displaying the highest and those in cluster III displaying the lowest expression level across the 20 tissues (Figure S6 and Table S8). We did not observe a consistent clustering of genes involved in the same process. Cluster I consists of seven genes (*BoCYP83A1*, *BoESP.2*, *BoGSTF9.1*, *BoGSTF9.2*, *BoGSTU20*, *BoGSTU13.1*, and *BoGSTU13.2*) involved in core structure synthesis, three genes (*BoGSH1.1*, *BoASA1.1,* and *BoASA1.2*) involved in cosubstrate pathways, two genes (*BoIIL1.1* and *BoIIL1.3*) involved in side-chain elongation, and one gene (*BoESP.2*) involved in GSL degradation. Both clusters II and III include a large number of genes involved in diverse phases/pathways, which showed extensive gene expression variation among different morphotypes and different tissues. For example, two genes (*BoESM1* and *BoPYK10.1*) involved in GSL degradation in cluster III were extremely highly expressed in roots in all the five morphotypes but remarkably lowly expressed in all other tissues. Only six genes (*BoCYP79C1*, *BoCYP79C2.1*, *BoMAM3.1*, *BoMYB118*, *BoNIT1;2;3,* and *BoSD1.3*) were not expressed in any tissue. The remaining 147 genes were all differentially expressed either between tissues or between morphotypes. This is well demonstrated by the heatmap constructed using *z*-scored TPM values (Figure 2). It is also clearly shown that many GSL genes were expressed at a higher level in roots than in other tissues for broccoli, kohlrabi, and white cabbage. Interestingly, those are generally not the same sets of genes and differ between the three mentioned morphotypes. In cauliflower, many genes were highly expressed in both roots and florets. For kale, and unlike the other four morphotypes, only a few genes were highly expressed in roots as compared to the other tissues.

### 3.3 Correlation between Gene Expression and GSL Levels

**Figure 3:**
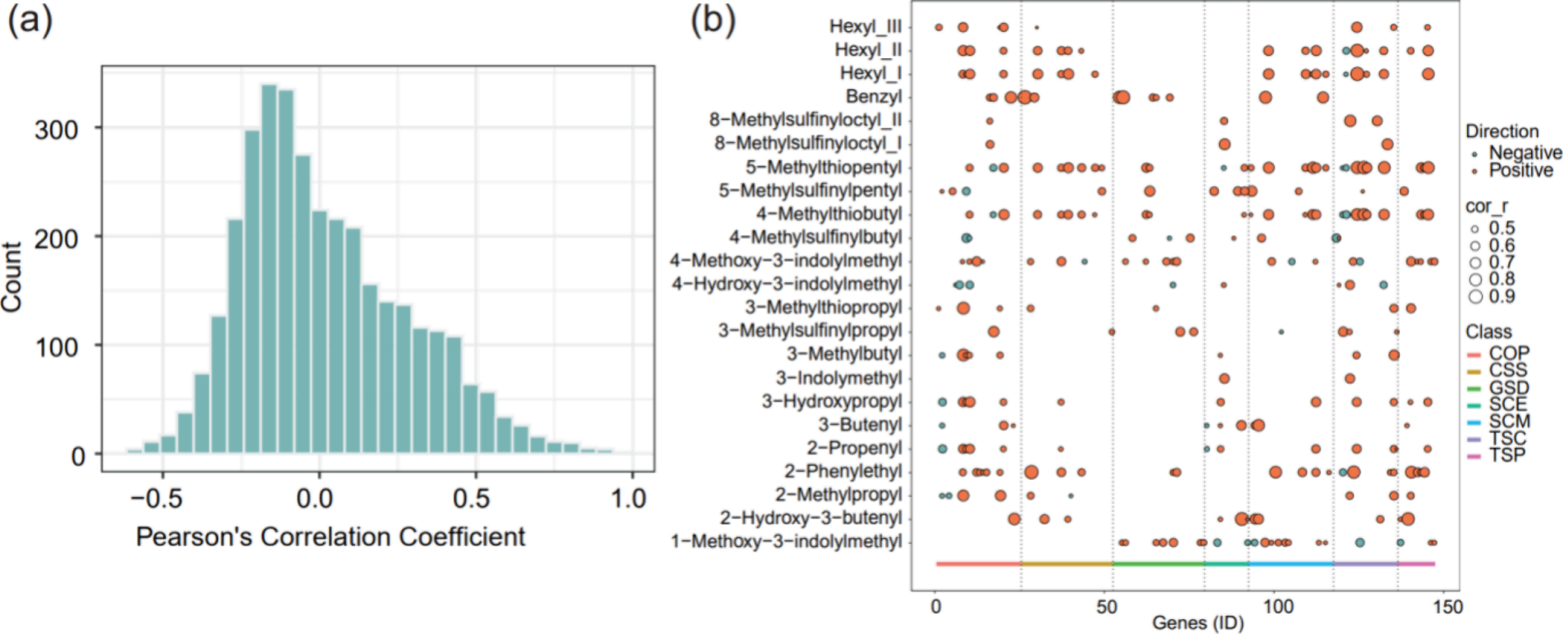
Pearson’s correlation analysis between GSLs and their related genes. (a) Distribution of Pearson’s correlation coefficient. (b) Significantly (*P* < 0.05) correlated GSLs and genes. Genes are classified based on their involvement in different processes/phases as shown in Figure 2. See Table S5 for the source data (abbreviations: COP: cosubstrate pathways, CSS: core structure synthesis, GSD: GSL degradation, SCE: side-chain elongation, SCM: side-chain modification, TSC: transcriptional components, and TSP: transporters).

To examine the correlation between the relative accumulation of each GSL and the expression of GSL related genes in *B. oleracea*, we performed a Pearson correlation analysis across all the 20 tissues. Out of the total 3381 combinations (147 expressed genes × 23 accumulating GSLs), 289 (8.55%) of them showed significant positive (251 combinations, *r* = 0.44–0.95, *P* < 0.05) or negative (38 combinations, *r* = −0.61 to −0.45, *P* < 0.05) correlations, which involve all the 23 GSLs and 109 genes (Figure 3a,b). We found that two white cabbage signature GSLs, 5-methylthiopentyl and 4-methylthiobutyl, were significantly correlated with the highest (28 genes) and second highest (26 genes) number of genes, respectively. The kale signature GSL 2-phenylethyl was significantly correlated with 23 genes (Tables S5 and S6). Forty-five out of the 109 genes were significantly correlated with a single GSL (Table S6). We also identified some genes that were strongly correlated with diverse GSLs (Figure 3b, Tables S5 and S6). For example, *BoAPR2*, a homologue of *AtAPR2* that is assumed to be involved in GSL cosubstrate pathways, was strongly correlated with eight aliphatic, one aromatic, and one indolic GSLs. *MYB122* is identified as a transcription factor that is needed for indolic GSL biosynthesis in *Arabidopsis*.^35^ Interestingly, in our morphotype/tissue samples, we discovered significant positive correlations between *BoMYB122* and eight aliphatic rather than indolic GSLs. As several genes are present in multiple copies (paralogues) with likely identical functions, we also performed the above correlation analysis after pooling the TPM values for paralogous GSL genes in *B. oleracea* (Figure S7). This resulted in a total of 167 gene–GSL combinations that showed significant correlation involving all the 23 GSLs and 62 pools of paralogous genes. Together, these genes that show significant correlation with GSLs may play an important role in regulating GSL biochemical pathways and thus GSL composition.

**Figure 4:**
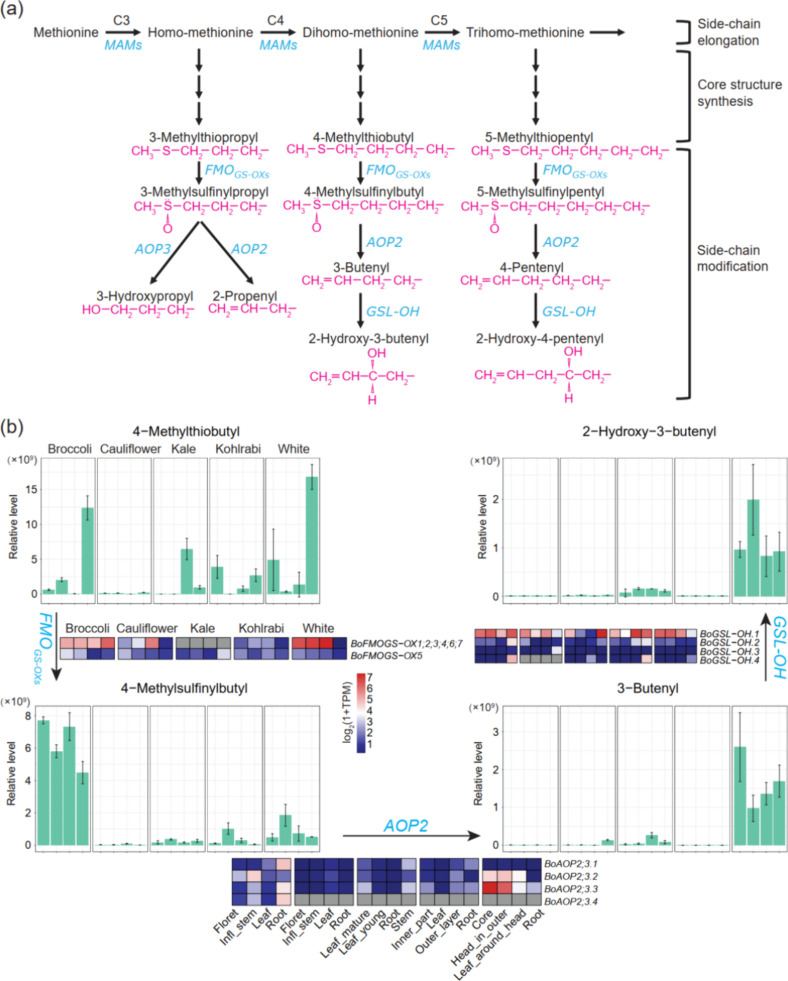
Aliphatic GSL biosynthesis pathway. (a) A genetic model of the biosynthesis of aliphatic GSLs with different chain length. The figure was adapted from Padilla et al.^36^ (b) C4 aliphatic GSL profiles and expression levels of related genes in different tissues and morphotypes. The bar charts show the relative abundance of individual C4 GSLs in respective tissues and morphotypes. Error bars indicate standard deviation (*n* = 3 biological replicates). Heatmaps show gene expression levels. Blue and red colors are used to represent low and high expression levels, respectively. Gray color denotes that the gene is not identified in the corresponding morphotype. In all the bar charts and heatmaps, samples are displayed in the same order. Note: *BoAOP2;3.3* is *BoAOP2*.

We then focused on specific genes putatively involved in side-chain modification processes in aliphatic GSL biosynthesis to investigate the correlation between gene expression and GSL levels (Figure 4a), namely, *FMO*_*GS-OX*_, *AOP* and *GSL-OH* genes, and the methionine-derived C3, C4, and C5 aliphatic GSLs. We identified two paralogues of *FMO*_*GS-OX*_, four paralogues of *AOP,* and four paralogues of *GSL-OH* in the *B. oleracea* genome (Figure 4b and Figure S8) and detected four C3, four C4, and two C5 methionine-derived aliphatic GSLs (Figure S3). In the C3-GSL biosynthesis pathway, the enzyme encoded by the *FMO*_*GS-OX*_ gene converts 3-methylthiopropyl into 3-methylsulfinylpropyl (Figure 4a). We observed the highest abundance of 3-methylthiopropyl and the lowest abundance of 3-methylsulfinylpropyl in roots of cauliflower, kale, and white cabbage (Figure S8a); in the same samples, the gene expression levels of the *FMO*_*GS-OX*_ paralogues were relatively low (although one paralogue was not identified in kale) (Figures S8a and S9), whose pattern may thus explain the corresponding relatively high and low levels of 3-methylthiopropyl and 3-methylsulfinylpropyl, respectively. This result suggests a direct relation between expression levels of *FMO*_*GS-OX*_ paralogues and the relative abundance of 3-methylthiopropyl and 3-methylsulfinylpropyl. In the next step of this pathway, *AOP2* and *AOP3* convert 3-methylsulfinylpropyl into 2-propenyl and 3-hydroxypropyl, respectively. The relative levels of both GSLs are highest in roots of cauliflower, kale, and white cabbage (Figure S8a). However, we did not observe any *AOP* paralogue displaying a higher expression level in roots than in other tissues of these three morphotypes (Figure S10). On the contrary, their expression was almost absent or relatively low in all cauliflower and kale tissues and relatively high for two out of three paralogues in the nonroot tissues of white cabbage. With regard to C4-GSLs, we observed a high accumulation of 4-methylsulfinylbutyl, whereas 3-butenyl was undetectable in all broccoli tissues (Figure 4b). However, *AOP* paralogues were expressed in several broccoli tissues including roots (Figure S10). 3-Butenyl was also low in cauliflower, kohlrabi, and kale, whereas it highly accumulated in all white cabbage tissues tested. This was related to a high expression of *AOP’s* in white cabbage compared to lower levels in all other morphotypes. In the next step of this pathway, *GSL-OH* converts 3-butenyl into 2-hydroxy-3-butenyl (Figure 4). Both these two compounds highly accumulated in white cabbage in all tissues and were not detectable in the other four morphotypes (Figure 4b). Interestingly, one paralogue of *BoGSL-OH* (*BoGSL-OH.1*) had a high expression level in all the five morphotypes (Figure S11). Besides the C4-GSL 4-methylsulfinylbutyl, broccoli also accumulated a relatively high level of the C5-GSL 5-methylsulfinylpentyl in all its tissues, whereas this compound was hardly detectable in any tissue of the four other morphotypes (Figure S8b); its conversion product 4-pentenyl GSL was not detectable in any of the five morphotypes. Together, these observations suggest that expression levels of *AOP* and *GSL-OH* genes cannot explain the relevant GSL variation.

### 3.4 Nonfunctional *AOP2* in Broccoli

*AOP2* catalyzes the conversion of 3-methylsulfinylpropyl, 4-methylsulfinylbutyl, and 5-methylsulfinylpentyl GSLs to the corresponding alkenyl GSLs of 2-propenyl, 3-butenyl, and 4-pentenyl, respectively^37,38^ (Figure 4a). It has been reported that broccoli harbors a nonfunctional *AOP2* allele, which results in the accumulation of 4-methylsulfinylbutyl.^39^ Also, in our study, both 4-methylsulfinylbutyl and 5-methylsulfinylpentyl levels were relatively high in broccoli (Figure 4b and Figure S8b), whereas their conversion products, 3-butenyl and 4-pentenyl GSL, respectively, were not detectable in broccoli (Figure 4b and Figure S8b). Based on sequence homology with *ArabidopsisAOP*s, we found three copies of *AOP* that are present in each of the five *B. oleracea* genomes; as *AOP2* and *AOP3* are highly homologous, it is difficult to define the function of the three *AOP* paralogues. Because white cabbage accumulated large amounts of 3-butenyl GSL in all its tissues (Figure 4b) and *BoAOP2;3.2* and *BoAOP2;3.3* are the only two *AOP*s that were expressed in white cabbage (Figure S10), it is suggested that *BoAOP2;3.2* or *BoAOP2;3.3* represents *BoAOP2.* Although *BoAOP2;3.2* and *BoAOP2;3.3* are clearly expressed in both broccoli and white cabbage (Figure S10), this does not lead to the expected accumulation of the enzyme product 3-butenyl GSL in broccoli, suggesting that *BoAOP2* is not functional in broccoli whereas it is so in white cabbage.

**Figure 5:**
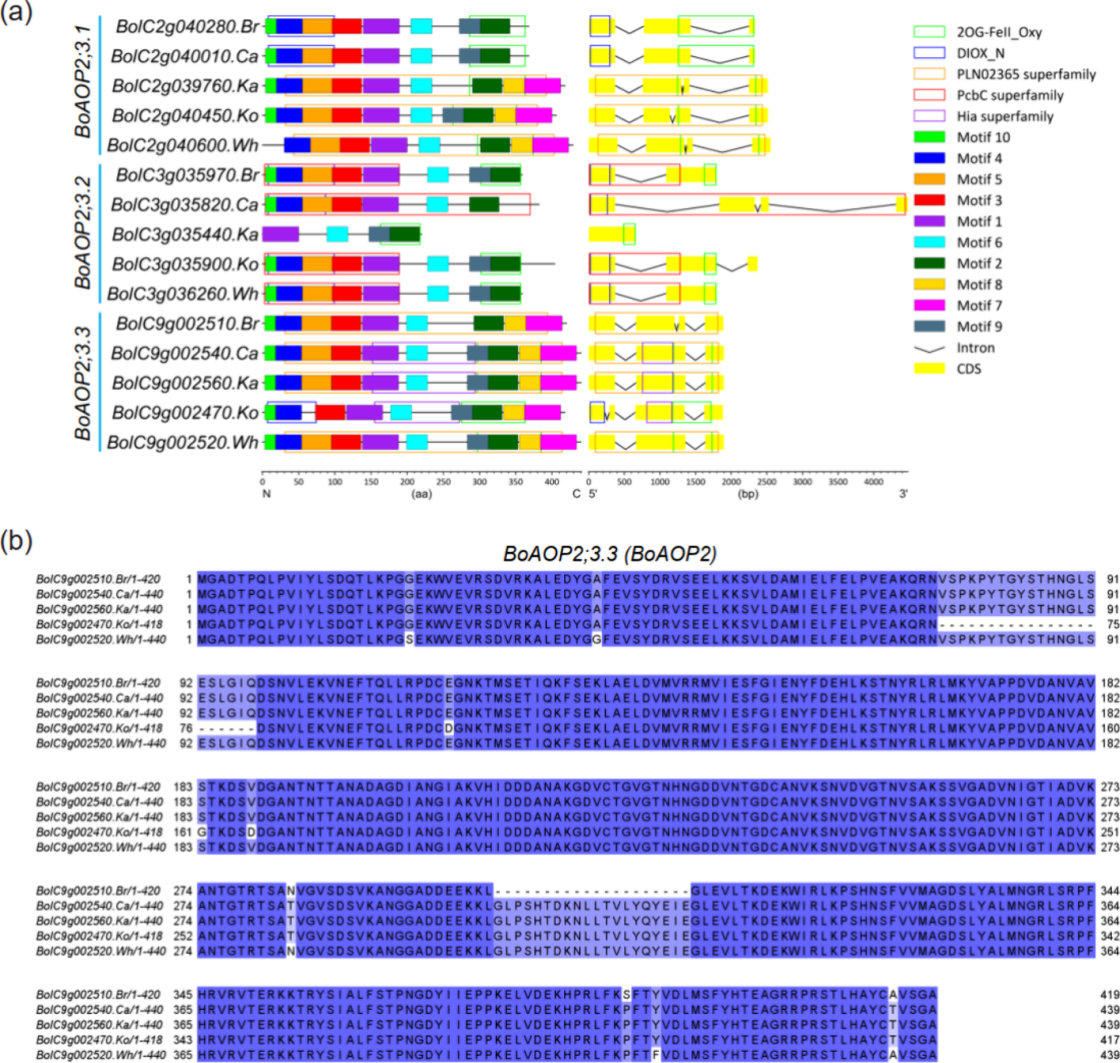
*AOP*s in five *B. oleracea* genomes. (a) Diagram of the motif, domain, and gene structure of *AOP* genes in *B. oleracea*. Different colored squares and boxes represent different motifs and domains, respectively. (b) Multiple alignments of the *BoAOP2* (*BoAOP2;3.3*) amino acid sequences.

To better understand the underlying genetic factor that may cause the nonfunctional *AOP2* specifically in broccoli, we compared the gene structure, motifs, and domains of 15 *BoAOP*s, i.e., the three *AOP* paralogues that were present in each of the five morphotypes. The gene lengths of both *BoAOP2;3.1* (2330–2553 bp) and *BoAOP2;3.3* (1883–1898 bp) homologues were similar among the five *B. oleracea* morphotypes but more variable for the *BoAOP2;3.2* (660–4479 bp) homologues (Figure 5a). The gene structure varied among the 15 *BoAOP*s, with most genes having three to four exons (Figure 5a). Accordingly, the motif compositions also varied among the 15 *BoAOP*s. All their encoded proteins contain three conserved motifs (motifs 1, 2, and 6), whereas other motifs varied between the 15 genes. We identified a total of five conserved domains across the 15 encoded proteins, and each *BoAOP* contained up to three domains (Figure 5a). Interestingly, the conserved 2OG-FeII_Oxy domain at the C-terminal was present in all *BoAOP*s except for two, i.e., broccoli *BoAOP2;3.3* (*BolC9g002510.Br*) and cauliflower *BoAOP2;3.2* (*BolC3g035820.Ca*). This protein domain is known to be essential for 2-oxoglutarate/Fe(II)-dependent dioxygenase activity, which is associated with an important class of enzymes that mediate a variety of oxidative reactions.^38,40^ The absence of the 2OG-FeII_Oxy domain in *BoAOP2;3.3* specifically in broccoli further suggests that *BoAOP2;3.3* represents *BoAOP2*. The gene structure comparison and multiple alignments of the amino acid sequences of the five *BoAOP2*’s clearly showed a novel intron in broccoli between exon 2 and exon 3 (Figure 5a,b), which possibly results in the absence of the 2OG-FeII_Oxy domain of its encoded enzyme. The amino acid sequence in this region is identical in the other four morphotypes, suggesting that *BoAOP2* is functional in these four morphotypes. Whereas the relative level of 3-butenyl GSL is high in white cabbage, it is very low in any tissue of cauliflower, kale, and kohlrabi; this can be due to either the low expression of *BoAOP2* or the lack of its precursor 4-methylsulfinylbutyl GSL.

### 3.5 Transposable Element Insertions Result in Long and Repeat-Rich Intronic Sequences in *BoMAM3.2*

**Figure 6:**
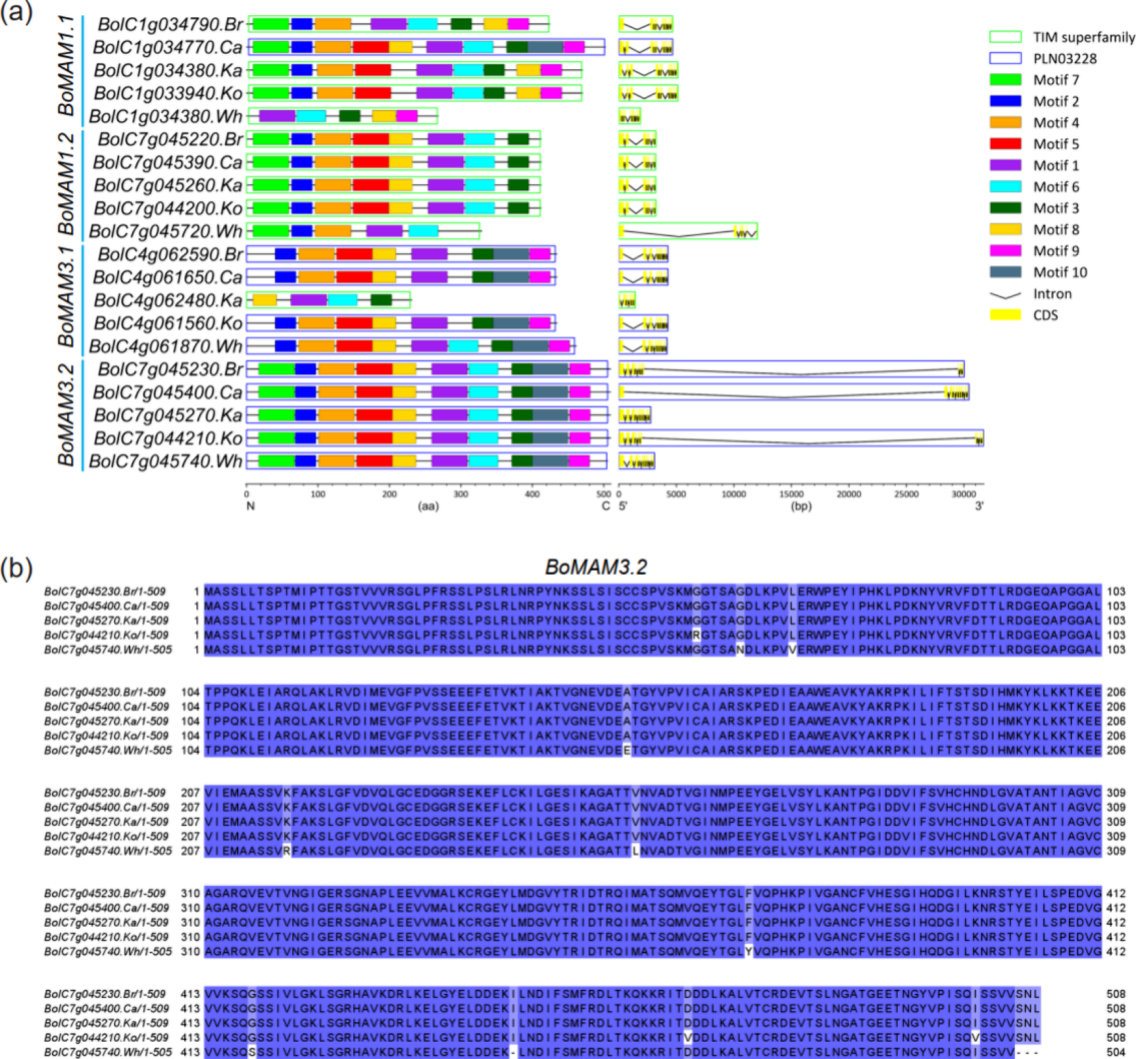
*MAMs* in five *B. oleracea* genomes. (a) Diagram of motif, domain, and gene structure of *MAM* genes in *B. oleracea*. Different colored squares and boxes represent different motifs and domains, respectively. (b) Multiple alignments of the *BoMAM3.2* amino acid sequences.

In *A. thaliana*, the *MAM* genes encode enzymes involved in chain elongation and produce GSLs with diverse chain lengths during the biosynthesis of methionine-derived GSLs.^41^*MAM1* catalyzes the condensation reaction of the first two elongation cycles, whereas *MAM3* is considered to contribute to the generation of all GSL chain lengths.^42^ We identified two paralogues of *MAM1* and two paralogues of *MAM3* in each of the five *B. oleracea* morphotypes. Structures and motifs of *MAM* genes in these genomes were then analyzed. Most *MAM* genes shared conserved gene structures (Figure 6a). From these 20 *MAMs*, three (white cabbage *MAM1.1* and *MAM1.2*, and kale *MAM3.1*) lost five to six conserved motifs and were thus heavily differentiated from the other homologues. We only identified two conserved domains across the 20 *BoMAMs* using the MEME tool.^29^ Interestingly, 9 out of 10 MAM1 proteins contain the TIM superfamily domain, and 9 out of 10 MAM3 proteins contain the PLN03228 domain, with each MAM1 and MAM3 having one protein displaying the opposite pattern (a cabbage *MAM1.1* and a kale *MAM3.1*) (Figure 6a).

The five proteins of *BoMAM3.2* have identical motif compositions, with each containing all 10 motifs. Accordingly, multiple alignments of the amino acid sequences of five *BoMAM3.2* homologues displayed remarkably high sequence similarity (Figure 6b). However, these genes strikingly varied in length, ranging from 2795 to 31,732 bp. By comparing the structure of the five *BoMAM3.2* homologues, we found three extremely long introns (27,317–29,020 bp) in broccoli, cauliflower, and kohlrabi. The predicted structures of these five *BoMAM3.2* genes are well supported by mRNA-seq evidence (Figure S13). Interestingly, we observed that a few mRNA-Seq reads could still be mapped to the three large intronic regions (Figure S13). A closer inspection of the three introns indicates that they were composed of many transposable elements (TEs), especially DNA transposons (Table S7). For example, we found a 2.8 kb DTC in broccoli and cauliflower and a 3.6 kb DTC in cauliflower and kohlrabi. Instead of introducing new introns, these TEs were inserted in different existing introns in the three *BoMAM3.2* genes. Gene expression profiles showed that *BoMAM3.2* was highly expressed in all five genomes in varying degrees, whereas the expression of its paralogue *BoMAM3.1* was hardly detectable in any morphotype (Figure S12). Also, most *BoMAM1* (*BoMAM1.1* and *BoMAM1.2*) genes were hardly expressed, except for low expression levels in broccoli and kohlrabi roots and in all white cabbage tissues. Although broccoli plants did not accumulate any C3 aliphatic GSL, they accumulated 4-methylsulfinylbutyl and 5-methylsulfinylpentyl GSLs (Figure 4b and Figure S8), which points to a functional *BoMAM3.2* (as *BoMAM3.1*, *BoMAM1.1,* and *BoMAM1.2* are not or very low expressed) gene converting all C3 GSLs to longer-chain GSLs. We also observed several long-chain aliphatic GSLs accumulating in the other morphotypes, such as 8-methylsulfinyloctyl GSL in both cauliflower and kale and possibly the less well described hexyl_III GSL in kale (Figure S3), suggesting that *BoMAM3.2* is also active in the other morphotypes. These TE insertion activities in *BoMAM3.2* may modify the expression level of the encoded gene.

## 4 Discussion

Glucoraphanin (4-methylsulfinylbutyl GSL) and glucoiberin (3-methylsulfinylpropyl GSL) are the two most desirable GSLs, in view of the nutritional value of their breakdown products.^15,43^ Among the five *B. oleracea* morphotypes, only broccoli accumulated relatively high levels of glucoraphanin in all its tissues (Figure S3b), whereas glucoiberin was relatively high in leaves and stems of cauliflower, kale, and white cabbage but was undetectable in broccoli (Figure S3a). In contrast to these two wanted GSLs, progoitrin (2-hydroxy-3-butenyl GSL) is unwanted because it can be hydrolyzed into oxazolidine-2-thione, which causes goiter and other harmful effects in mammals.^44^ Interestingly, progoitrin was relatively high in all tissues of white cabbage, whereas it was not detectable in any of the sampled tissues of the other four morphotypes (Figure S3b). Previously, Yi et al.^15^ showed that progoitrin was absent in florets in one of the three cauliflower genotypes investigated. Wang et al.^43^ found comparatively higher progoitrin in commercial broccoli genotypes than in inbred lines. These suggest that GSL content also varies between genotypes of the same morphotype in *B. oleracea*. To breed varieties with tailored GSL content, the information with regard to which genotype and organ these desired and unwanted compounds are accumulated at which quantitative level is vital for parental selection.

Several reasons could possibly explain why the vast majority (91.45%) of GSL–gene combinations (3092 out of 3381 combinations) do not show correlation between GSL relative abundance and gene expression level (Figure 3). First, the GSL biosynthesis pathway is very complex, with many compounds being produced that can either accumulate or convert into derived compounds (like the side-chain modification steps) or even convert into a breakdown product (like the ITCs, nitriles, and indoles). As a consequence, the relative abundance of an intermediate GSL does not necessarily show a correlation with the expression level of its regulating genes. A direct correlation between the activity of a structural enzyme (presuming this activity is fully regulated by its gene expression level) and its product can however only be expected if the next step in a pathway is absent or highly limiting. Second, GSL transporters have been identified and experimentally verified,^18,45^ which establish dynamic GSL patterns between source and sink tissues in *Arabidopsis*. The function of transporters is to import GSL from the apoplastic apace to the symplast.^18,45^ They should be present in both sink and source tissues. The long-distance transport is via the vascular system. Thus, GSL compounds accumulated in a specific tissue do not necessarily mean that they are originally produced in this tissue, and so are not directly correlated to the expression level of a responsible biosynthetic gene in that specific tissue. Third, the biosynthesis and regulation of GSLs are well studied in the model plant *A. thaliana*, whereas most studies in *Brassica* crops are based on this reference pathway.^6^ The function of these presumed genes in *Brassica* has to be further verified. Because of the whole genome triplication event in *Brassica*, many genes are present in multiple copies, which may display different expression patterns. Because of sequence divergence during evolution, it is also difficult to know which copy is functional. In addition, nonfunctional genes can still be quantified as expressed by mRNA-Seq data, such as the nonfunctional *AOP2* in broccoli. Lastly, Yu et al.^46^ suggested that much of the regulation of metabolite levels in tea may occur not only at the transcriptional level but at multiple levels, such as transcriptional, post-transcriptional, translational, post-translational, and epigenetic levels, which also seems possible for GSLs in *Brassica* crops. Despite all these challenges, we still detected 289 GSL–gene combinations with significant correlation between GSL relative abundance and gene expression level. Interestingly, several GSLs were correlated with the expression of many genes that were involved in different processes (like core structure synthesis pathway, side-chain elongation and modification, but also cosubstrate and transport and regulatory pathways). Unexpectedly, we discovered significant positive correlations between *BoMYB122* and eight aliphatic GSLs, whereas in *Arabidopsis,MYB122* is known to regulate indolic GSL biosynthesis. However, the study by Frerigmann and Gigolashvili^35^ demonstrated only a minor role of *MYB122* in indolic GSL regulation in *Arabidopsis* at standard growth conditions, with only an accessory role in indolic GSL regulation upon environmental challenges. In *B. oleracea*, the regulatory role of MYB122 in indolic GSL biosynthesis has not been experimentally tested. It is likely that the regulatory mechanism in *B. oleracea* differs from that in *Arabidopsis*, as suggested by the significantly positive correlations between *BoMYB122* and eight aliphatic GSLs. To better understand GSL biosynthesis and identify genetic loci controlling GSL production in *Brassica* crops, more extensive and in-depth studies, such as genome-wide association analysis of genomic SNPs, transcriptomic and GSL data from a large number of samples, the construction of gene regulatory networks, and mGWAS, will be needed.

In our study, we investigated the sequence divergence of two important GSL genes (*AOP2* and *MAM3*) among the five different *B. oleracea* morphotypes. *AOP2* is not functional in broccoli, which results in the accumulation of two main health-promoting compounds in this morphotype: 4-methylsulfinylbutyl (glucoraphanin) and 5-methylsulfinylpentyl (glucoalyssin). However, the functional *AOP2* in white cabbage converts 4-methylsulfinylbutyl into highly accumulated 3-butenyl. The functional divergence of *AOP2* between these morphotypes is likely attributed to their sequence divergence, with *AOP2* in broccoli lacking a conserved 2OG-FeII_Oxy domain. We found many TE insertion activities in one paralogue of *MAM3* (*BoMAM3.2*) in three morphotypes, which resulted in the extremely long (∼29 kb) and repeat-rich intronic sequences. As these insertions happen in existing introns and we identified long-chain aliphatic GSLs for which *MAM3* is responsible, the gene function is unlikely to have been altered. Cai et al.^47^ reported that TE insertions within introns tend to largely modify gene expression levels. We observed that this gene is differentially expressed among tissues and among morphotypes (Figure S12), and the TE insertion activities may have contributed to the expression difference between morphotypes. In addition, extremely long introns seem to be prevalent in plant genomes. For example, they have been found in *Arabidopsis*, with lengths larger than 5 kb or even 10 kb.^48^ Accordingly, Liu et al.^49^ reported that ginkgo possesses very long introns characterized by many repeat-element insertions, with 10% of its longest introns even greater than 100 kb. It is also reported that large introns are involved in regulating gene expression levels,^50^ probably through intron DNA methylation. Qin et al. conducted a comprehensive review of potential targets for current and future GSL metabolic engineering.^51^ They highlighted *AOP2* and *MAM3* as targets that were manipulated through overexpression or knockout experiments in *Arabidopsis*, leading to alteration in GSL profiles. Building upon this knowledge, our study contributes novel insights into the manipulation of these two genes to modulate GSL levels and composition specifically in *B. oleracea*.

In summary, we profiled GSLs and mRNA-Seq in roots, leaves, and the edible parts of five different *B. oleracea* morphotypes, revealing strong variations of GSL relative abundance and composition as well as GSL related gene expression. We found a total 289 GSL–gene combinations with significant correlation between GSL and gene expression level, which involve all the 23 GSLs and 109 related genes. We observed a nonfunctional *AOP2* in broccoli, which is related to the loss of a conserved 2OG-FeII_Oxy domain and results in the accumulation of the health-promoting compounds 4-methylsulfinylbutyl (glucoraphanin) and 5-methylsulfinylpentyl (glucoalyssin). We also found many TE insertions in one paralogue of *MAM3* gene in three genomes, affecting long-chain aliphatic GSLs.

## Data Availability

Raw sequencing reads for the mRNA-Seq data in this study have been deposited in NCBI under the accession number PRJNA982784 (https://www.ncbi.nlm.nih.gov/sra/?term=PRJNA982784). Relative levels of identified GSLs in different *B. oleracea* morphotypes and tissues can be found in Table S9.

## Supporting Information Available

(Figure S1) The five *B. oleracea* morphotypes and collected tissues for GSL extraction and RNA sequencing. (Figure S2) Principal component analysis (PCA) based on GSL data showing overall variation between the three biological replicates (20 samples × 3 biological replicates). (Figure S3) Relative quantity of individual GSL in four tissues of five *B. oleracea* morphotypes. (a) Methionine-derived aliphatic C3 GSLs. (b) Methionine-derived aliphatic C4 GSLs. (c) Methionine-derived aliphatic C5 GSLs. (d) Methionine-derived aliphatic C8 GSLs. (e) Branched-chain amino acid derived aliphatic GSLs. (f) Aliphatic hexyl GSLs. (g) Indolic GSLs. (h) Aromatic GSLs. The *Y* axis shows the peak surface area measured in LCMS for the indicated compound. Error bars indicate standard deviation (*n* = 3). (Figure S4) Distribution of GSL related genes in the five *B. oleracea* genomes. The number of GSL related genes in nonoverlapped 2-Mb windows was calculated. See Table S3 for source data. (Figure S5) PCA plots and cluster dendrogram based on mRNA-Seq data. In PCA plots, morphotypes from the top to bottom are broccoli, cauliflower, kale, kohlrabi, and white cabbage. Sample IDs in cluster dendrogram correspond to those used in PCA plots. Abbreviations in the PCA legends: BrLe: broccoli leaf, BrRo: broccoli root, BrIs: broccoli infl_stem, BrFl: broccoli floret, CaLe: cauliflower leaf, CaRo: cauliflower root, CaIs: cauliflower infl_stem, CaFl: cauliflower floret, KaLm: kale leaf_mature, KaLy: kale leaf_young, KaSt: kale stem, KaRo: ssskale root, KoLe: kohlrabi leaf, KoOl: kohlrabi outer_layer, KoIn: kohlrabi inner_part, KoRo: kohlrabi root, WhCo: white core, WhLh: white leaf_around_head, WhHo: white head_in_outer, and WhRo: white root. (Figure S6) Expression profiles for GSL related genes in four tissues of five *B. oleracea* morphotypes. Heatmaps were constructed using log2 transformed TPM values. Blue and red colors are used to represent low and high expression levels, respectively. Red arrows indicate nodes for the three clusters. Genes are classified based on their involvement in different processes/phases (abbreviations: COP: cosubstrate pathways, CSS: core structure synthesis, GSD: GSL degradation, SCE: side-chain elongation, SCM: side-chain modification, TSC: transcriptional components, and TSP: transporters). (Figure S7) Pearson’s correlation analysis between GSLs and their related genes. Gene expression profiles (TPM values) are pooled for different copies of paralogous genes in *B. oleracea*. (a) Distribution of Pearson’s correlation coefficient. (b) Significantly (*P* < 0.05) correlated GSLs and genes. Genes are classified based on their involvement in different processes/phases as shown in Figure 2 (abbreviations: COP: cosubstrate pathways, CSS: core structure synthesis, GSD: GSL degradation, SCE: side-chain elongation, SCM: side-chain modification, TSC: transcriptional components, and TSP: transporters). (Figure S8) C3 (a) and C5 (b) aliphatic GSL profiles and expression levels of related genes in different tissues and morphotypes. The bar charts show relative quantity of individual GSLs in respective tissues and morphotypes. Error bars indicate standard deviation (*n* = 3). Heatmaps show gene expression levels. Blue and red colors are used to represent low and high expression levels, respectively. Gray color denotes that the gene is not identified in the corresponding morphotype. In all the bar charts and heatmaps, samples are displayed in the same order. Note: *BoAOP2;3.3* is *BoAOP2.* (Figure S9) Gene expression analysis of *FMO*_*GS-OX*_ paralogues in four tissues in five *B. oleracea* morphotypes. The expression level was estimated using TPM values based on mRNA-Seq data. Error bars indicate standard deviation (*n* = 3). (Figure S10) Gene expression analysis of *AOP* paralogues in four tissues in five *B. oleracea* morphotypes. The expression level was estimated using TPM values based on mRNA-Seq data. Error bars indicate standard deviation (*n* = 3). Note: *BoAOP2;3.3* is *BoAOP2.* (Figure S11) Gene expression analysis of *GSL-OH* paralogues in four tissues in five *B. oleracea* morphotypes. The expression level was estimated using TPM values based on mRNA-Seq data. Error bars indicate standard deviation (*n* = 3). (Figure S12) Gene expression analysis of *MAM* paralogues in four tissues in five *B. oleracea* morphotypes. The expression level was estimated using TPM values based on mRNA-Seq data. Error bars indicate standard deviation (*n* = 3). (Figure S13) IGV snapshots showing mRNA-Seq alignments in *BoMAM3.2* genes from five *B. oleracea* morphotypes (top to bottom: broccoli, cauliflower, kohlrabi, kale, and white cabbage). In each snapshot, four tracks from top to bottom represent alignments in four different tissues (PDF)
(Table S1) Signature GSLs for the five *B. oleracea* morphotypes (Student–Newman–Keuls test with α = 0.05). (Table S2) GSL related genes identified in the five *B. oleracea* genomes. (Table S3) Position of GSL related genes identified in the five *B. oleracea* genomes. (Table S4) Summary of RNA-Seq data and statistics for read mapping. (Table S5) List of significantly correlated GSLs and related genes. (Table S6) The number of GSLs/genes that are significantly correlated with the given gene/GSL. (Table S7) TE annotations in the long intron of *MAM3* gene in three *B. oleracea* genomes. (Table S8) Gene expression (TPM values) of GSL related genes in sampled tissues. (Table S9) Relative quantities of GSLs in different *B. oleracea* morphotypes and tissues (XLSX)
