# Supplementary material for: Metabolomic and Transcriptomic Profiles in Diverse Brassica oleracea Crops Provide Insights into the Genetic Regulation of Glucosinolate Profiles
Source: J Agric Food Chem. 2024 Jul 8;72(28):16032–44. doi: 10.1021/acs.jafc.4c02932 (PMC11261609; doi:10.1021/acs.jafc.4c02932)
Supplement: Supplementary file 1 — jf4c02932_si_001.pdf [file jf4c02932_si_001.pdf]

Supporting Information

Metabolomic and transcriptomic profiles in diverse *Brassica oleracea* crops provide insights into genetic regulation of glucosinolate profiles

Chengcheng Cai[1,2], Ric C.H. de Vos[3], Hao Qian[1], Johan Bucher[1] and Guusje Bonnema[1,*]

[1] Plant Breeding, Wageningen University and Research, 6708 PB, Wageningen, The Netherlands

[2] State Key Laboratory of Vegetable Biobreeding, Key Laboratory of Biology and Genetic Improvement of Horticultural Crops of the Ministry of Agriculture and Rural Affairs, Sino-Dutch Joint Laboratory of Horticultural Genomics, Institute of Vegetables and Flowers, Chinese Academy of Agricultural Sciences, 100081, Beijing, China

[3] Bioscience, Wageningen University and Research, 6708 PB, Wageningen, The Netherlands

Email address: Chengcheng Cai, tsaicc@126.com; Ric de Vos, ric.devos@wur.nl; Hao Qian, qianh373@gmail.com; Johan Bucher, johan.bucher@wur.nl; Guusje Bonnema, guusje.bonnema@wur.nl

***Correspondance:** Guusje Bonnema (guusje.bonnema@wur.nl), Tel: +31 317484028

Table of Contents

Supplementary figures (Fig. S1-S13)

Supplementary tables (Table S1-S9) (Data shown in another excel file)

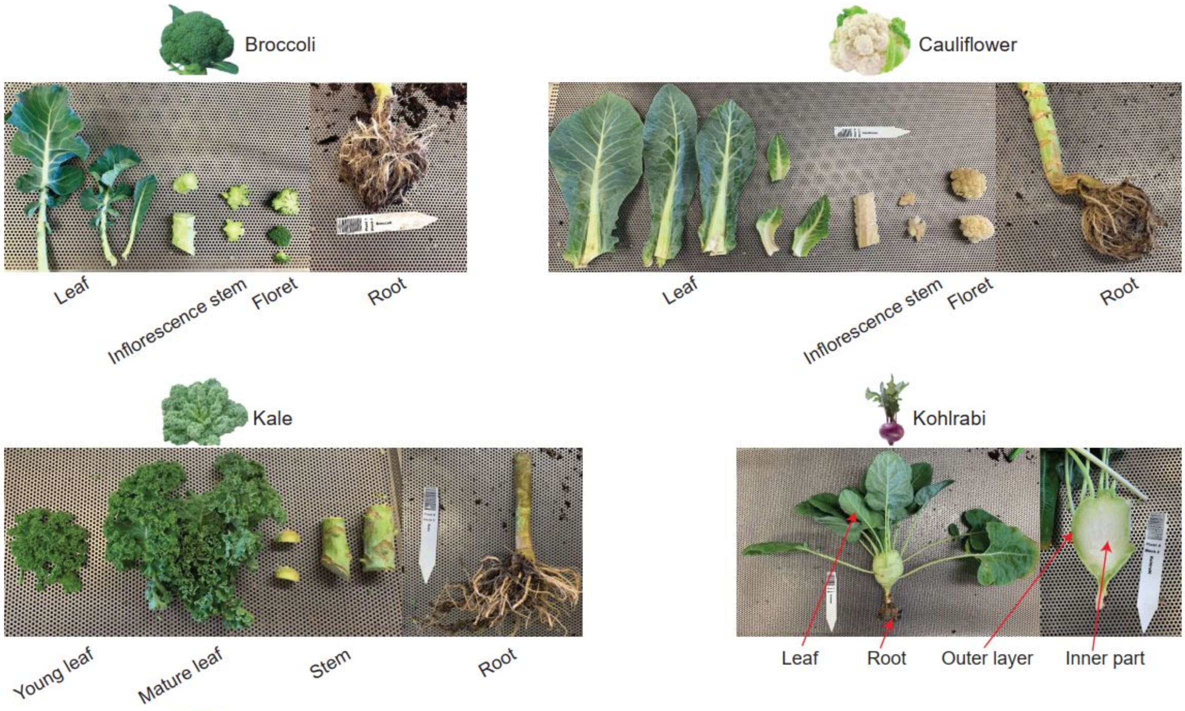

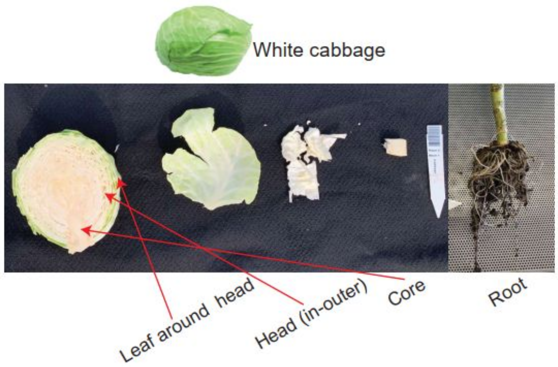

Sowing and harvest time

Morphotype	Sowing date	Harvest date	Days after sowing (DAS)
Broccoli	21-04-2020	21-07-2020	91
Cauliflower	21-04-2020	13-08-2020	114
Kale	21-04-2020	01-09-2020	133
Kohlrabi	29-04-2020	03-07-2020	65
White cabbage	21-04-2020	11-09-2020	143

Fig. S1 The five *B. oleracea* morphotypes and collected tissues for GSL extraction and RNA sequencing.

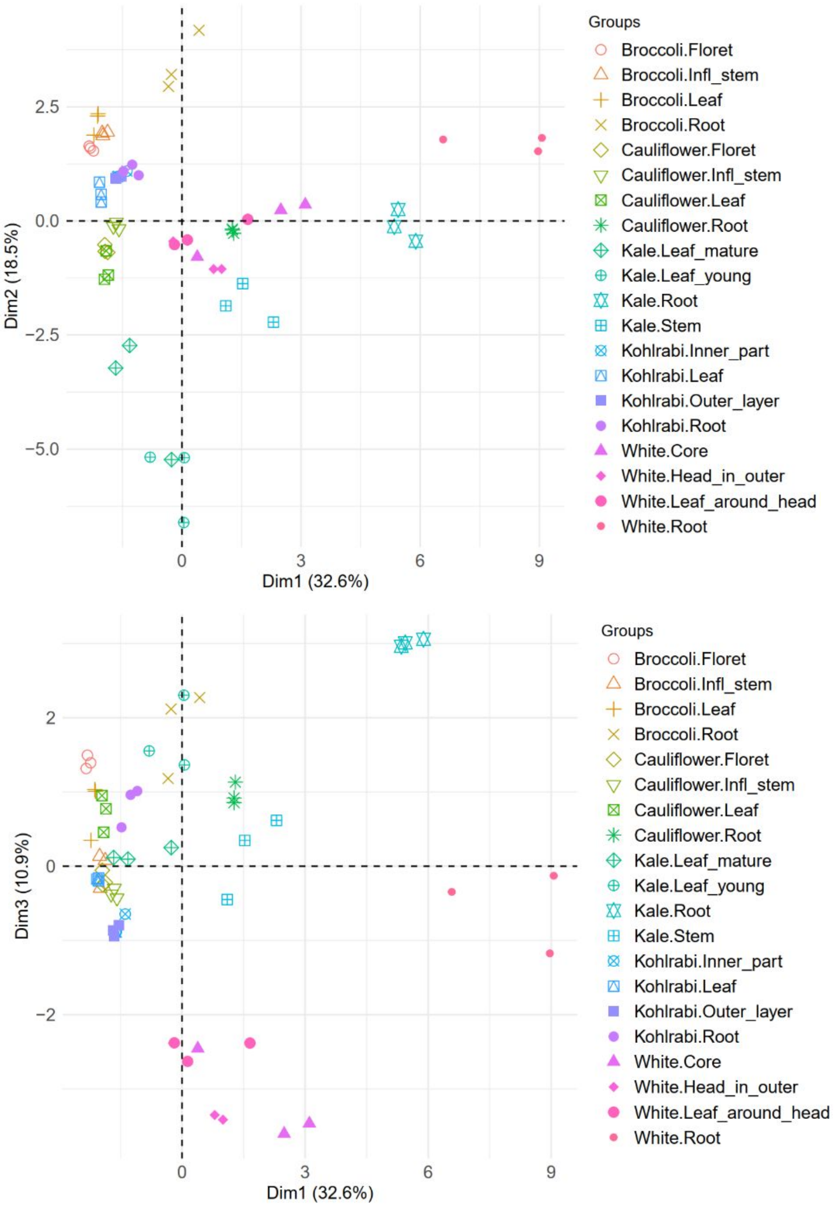

Fig. S2 Principal component analysis (PCA) based on GSL data showing overall variation between the three biological replicates (20 samples × 3 biological replicates).

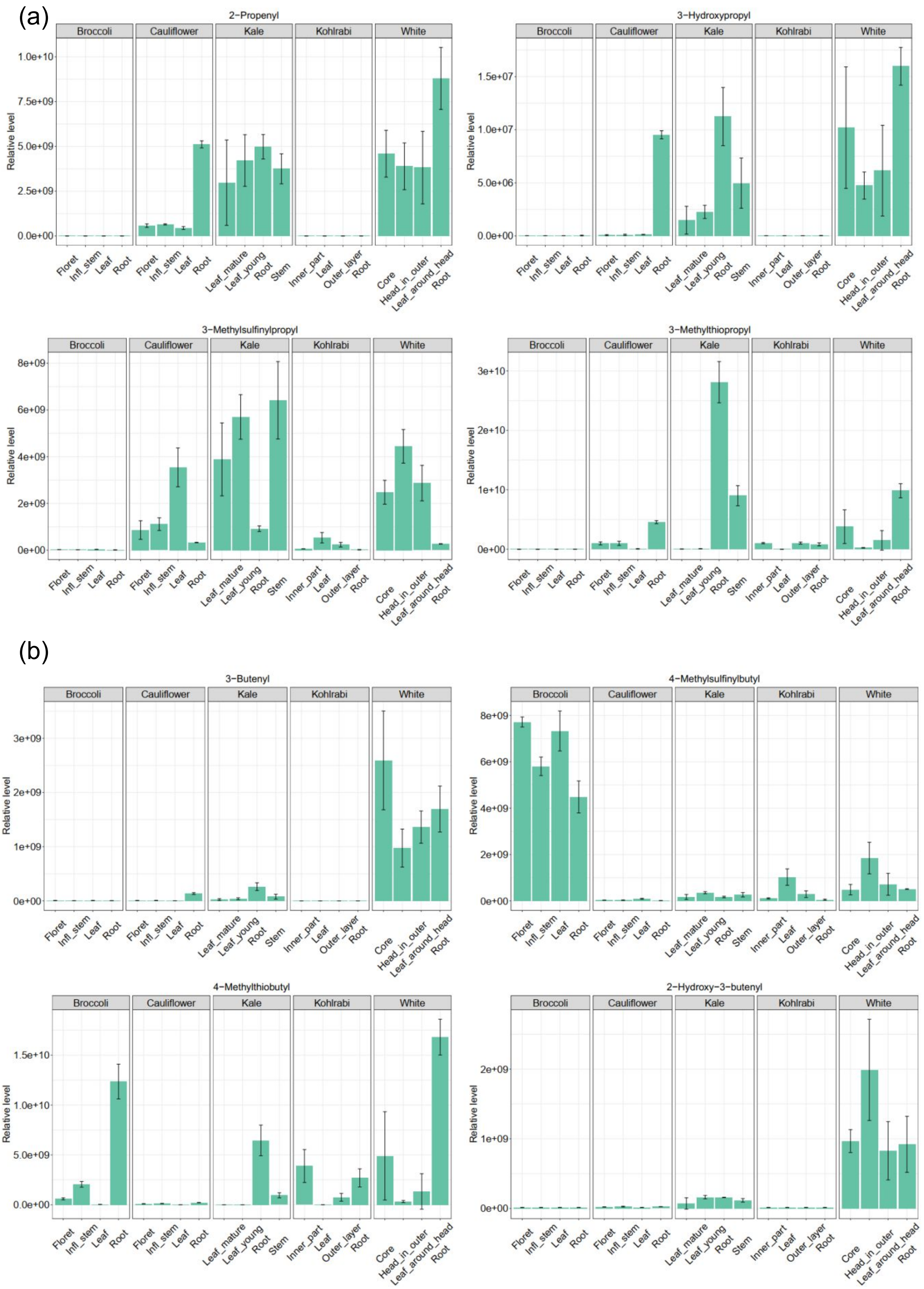

(a)
2−Propenyl
3−Hydroxypropyl
3−Methylsulfinylpropyl
3−Methylthiopropyl
(b)
3−Butenyl
4−Methylsulfinylbutyl
4−Methylthiobutyl
2−Hydroxy−3−butenyl
Broccoli
Cauliflower
Kale
Kohlrabi
White
Relative level
Floret
Infl_stem
Leaf
Root
Leaf_mature
Leaf_young
Stem
Inner_part
Outer_layer
Core
Head_in_outer
Leaf_around_head

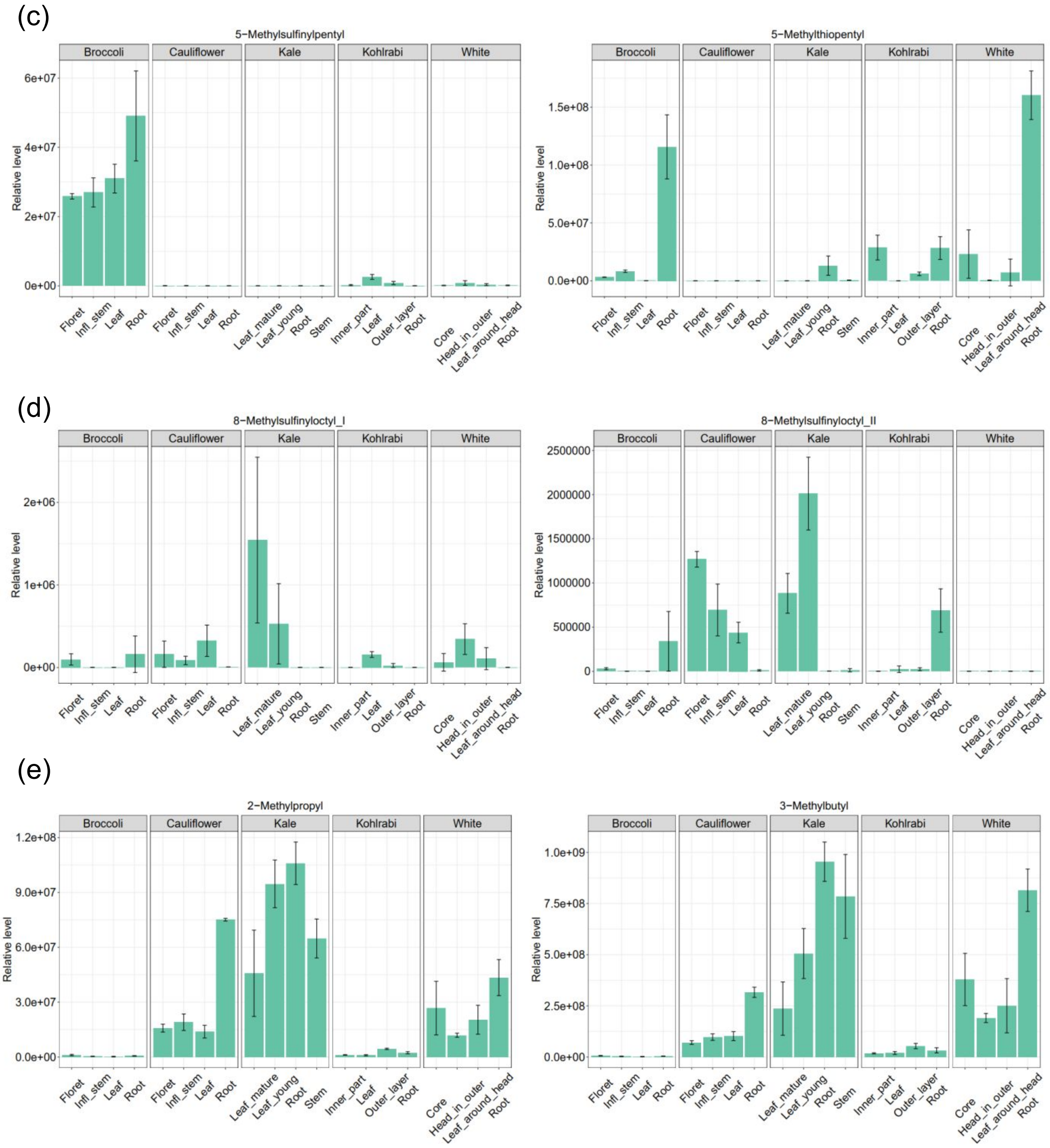
(c)
5−Methylsulfinylpentyl
5−Methylthiopentyl
(d)
8−Methylsulfinyloctyl_I
8−Methylsulfinyloctyl_II
(e)
2−Methylpropyl
3−Methylbutyl
Broccoli
Cauliflower
Kale
Kohlrabi
White
Relative level
Floret
Infl_stem
Leaf
Root
Leaf_mature
Leaf_young
Stem
Inner_part
Outer_layer
Core
Head_in_outer
Leaf_around_head
0e+00
2e+07
4e+07
6e+07
0.0e+00
5.0e+07
1.0e+08
1.5e+08
1e+06
2e+06
0
500000
1000000
1500000
2000000
2500000
3.0e+07
6.0e+07
9.0e+07
1.2e+08
2.5e+08
5.0e+08
7.5e+08
1.0e+09

(f)

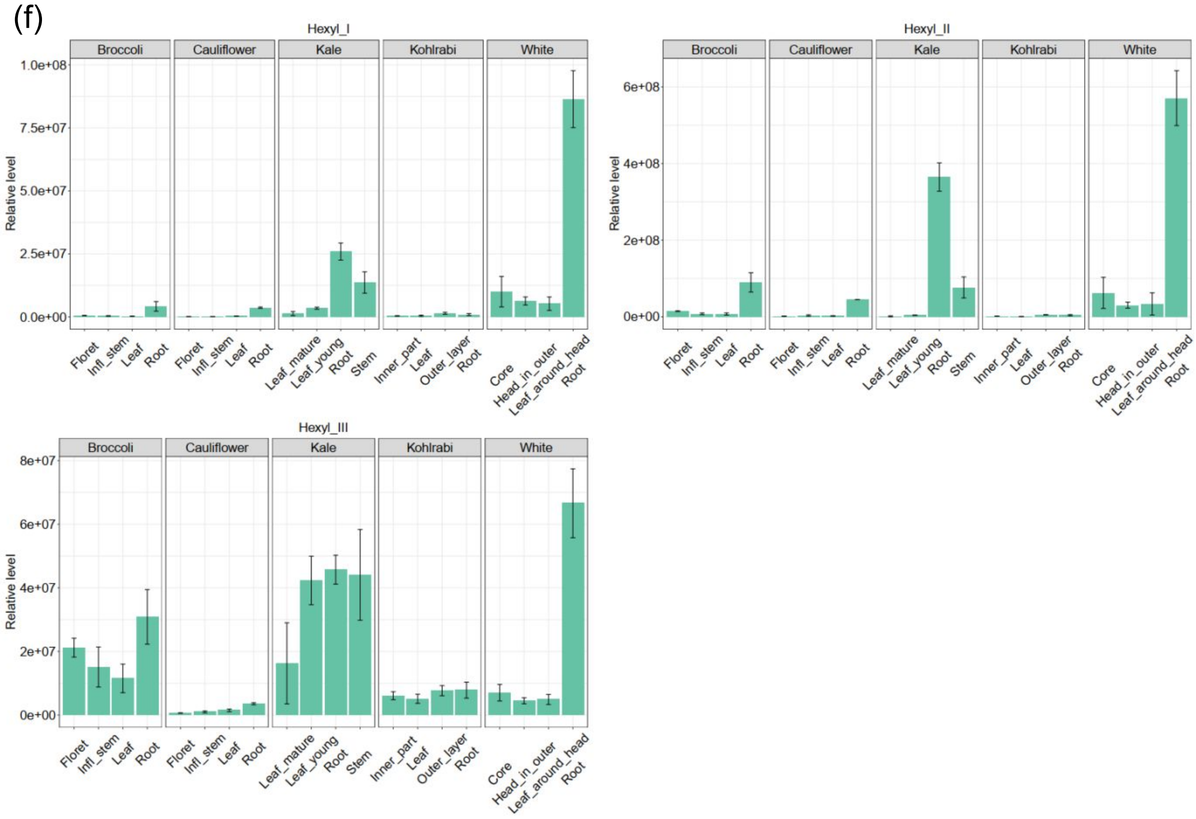

Hexyl_I
Hexyl_II
Hexyl_III
Broccoli
Cauliflower
Kale
Kohlrabi
White
Relative level
1.0e+08
7.5e+07
5.0e+07
2.5e+07
0.0e+00
6e+08
4e+08
2e+08
0e+00
8e+07
6e+07
4e+07
2e+07
0e+00
Floret
Infl_stem
Leaf
Root
Leaf_mature
Leaf_young
Stem
Inner_part
Outer_layer
Core
Head_in_outer
Leaf_around_head

(g)

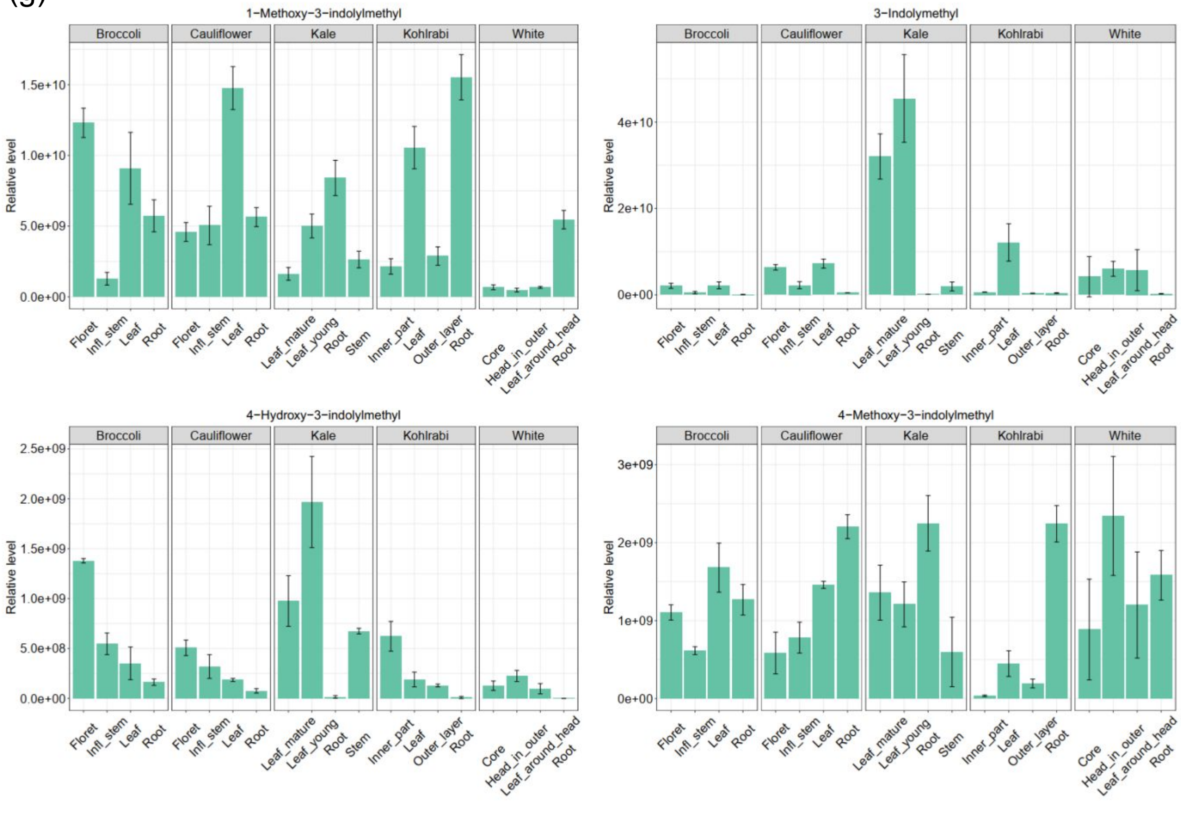

1−Methoxy−3−indolylmethyl
3−Indolymethyl
4−Hydroxy−3−indolylmethyl
4−Methoxy−3−indolylmethyl
Broccoli
Cauliflower
Kale
Kohlrabi
White
Relative level
1.5e+10
1.0e+10
5.0e+09
0.0e+00
4e+10
2e+10
0e+00
2.5e+09
2.0e+09
1.5e+09
1.0e+09
5.0e+08
0.0e+00
3e+09
2e+09
1e+09
0e+00
Floret
Infl_stem
Leaf
Root
Leaf_mature
Leaf_young
Stem
Inner_part
Outer_layer
Core
Head_in_outer
Leaf_around_head

(h)

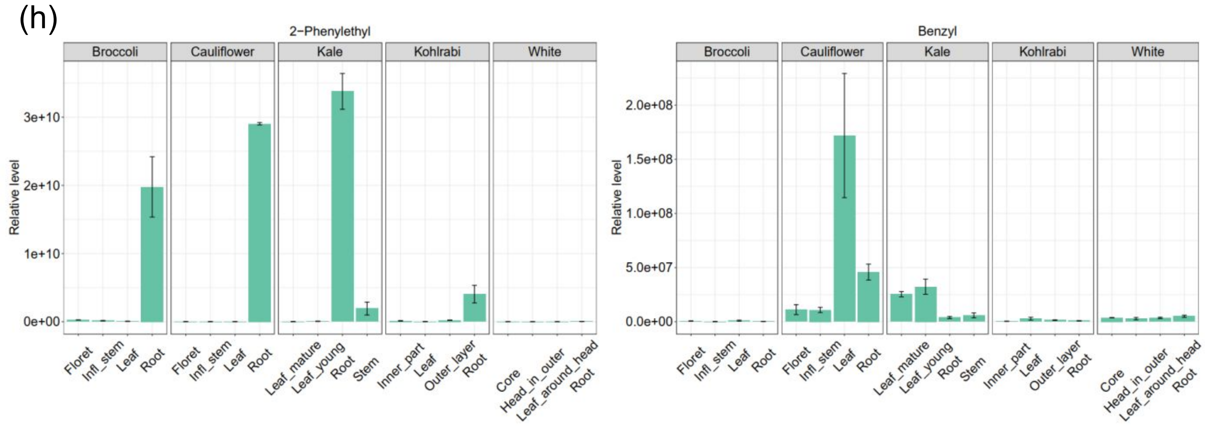

Fig. S3 Relative quantity of individual GSL in four tissues of five *B. oleracea* morphotypes. (a) Methionine derived aliphatic C3 GSLs. (b) Methionine derived aliphatic C4 GSLs. (c) Methionine derived aliphatic C5 GSLs. (d) Methionine derived aliphatic C8 GSLs. (e) Branched-chain amino acid derived aliphatic GSLs. (f) Aliphatic hexyl GSLs. (g) Indolic GSLs. (h) Aromatic GSLs. The Y-axis shows the peak surface area measured in LCMS for the indicated compound. Error bars indicate standard deviation (n = 3).

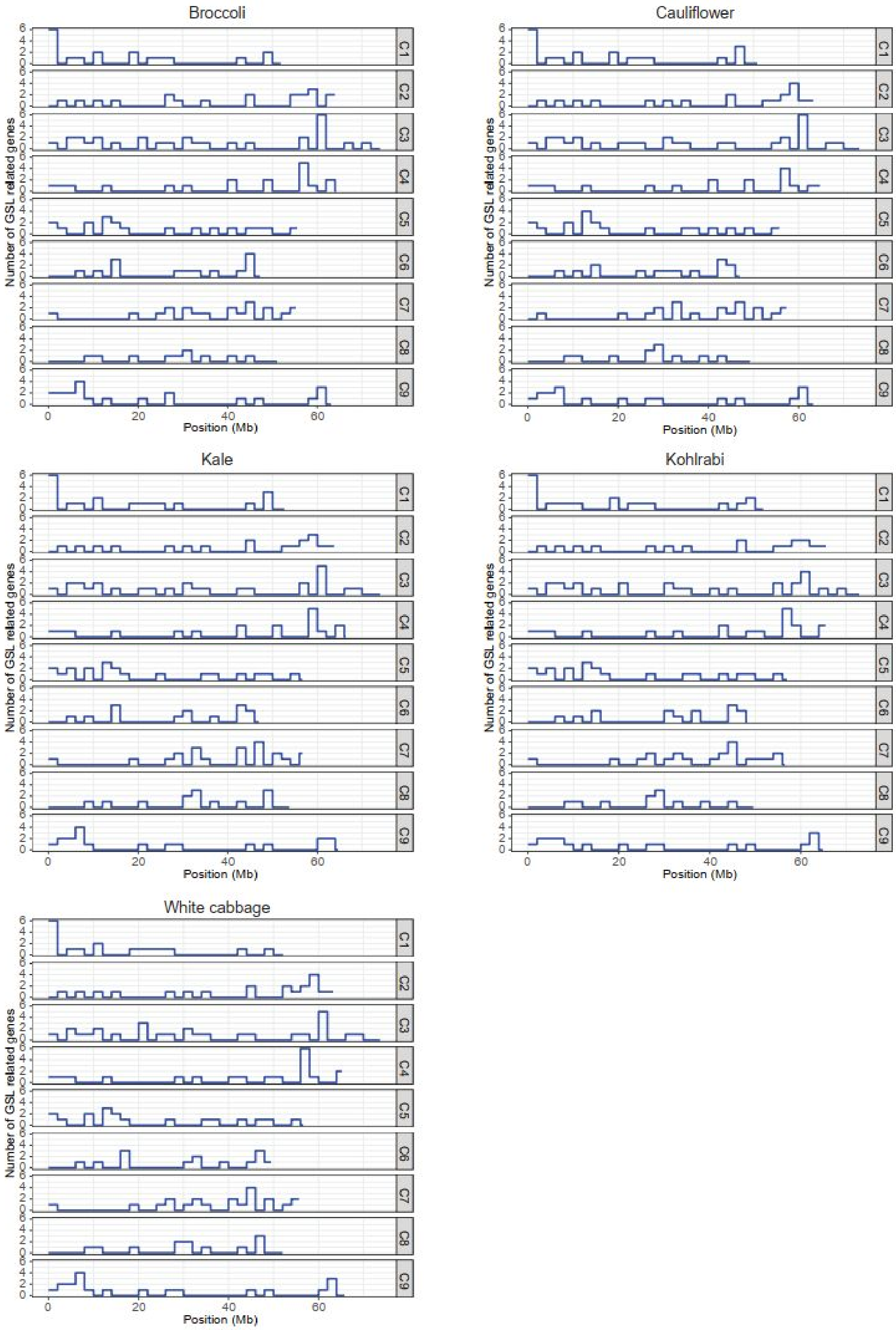

Fig. S4 Distribution of GSL related genes in the five *B. oleracea* genomes. The number of GSL related genes in non-overlapped 2-Mb windows was calculated. See Table S3 for source data.

Principal Component Analysis

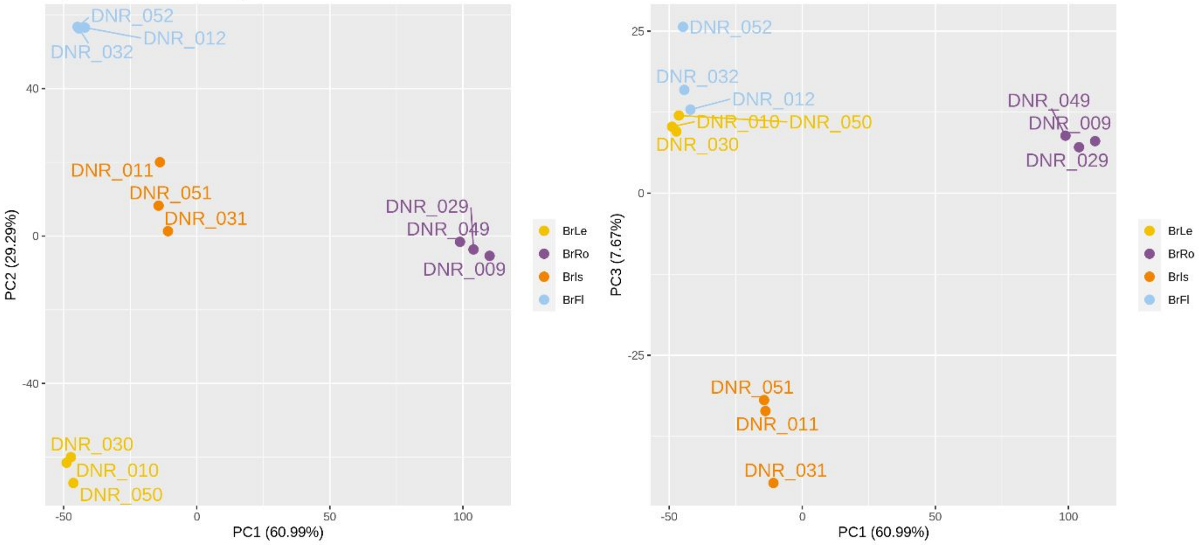

Principal Component Analysis

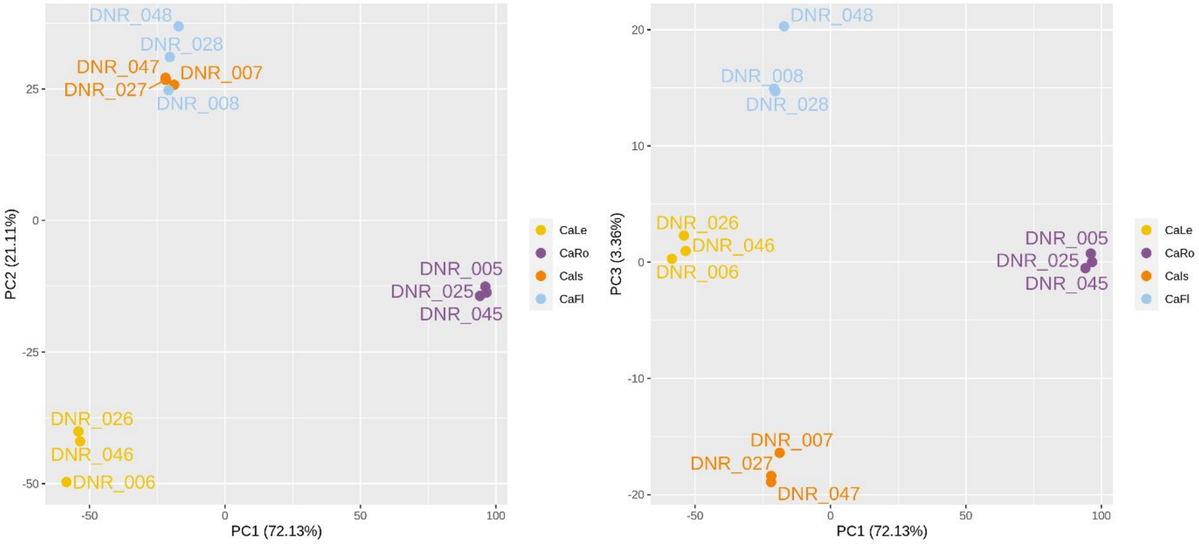

Principal Component Analysis

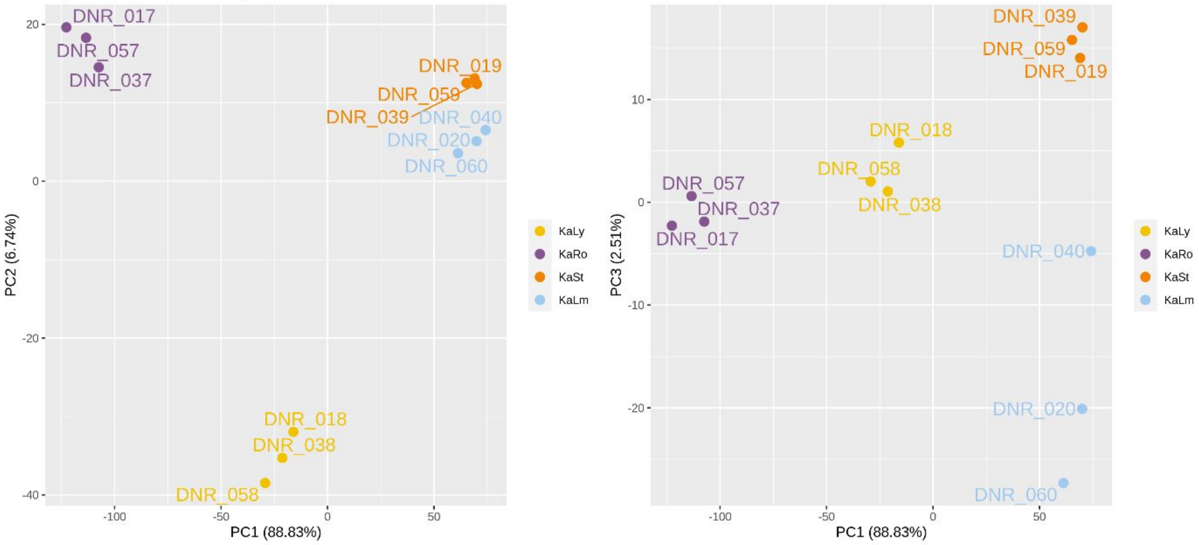

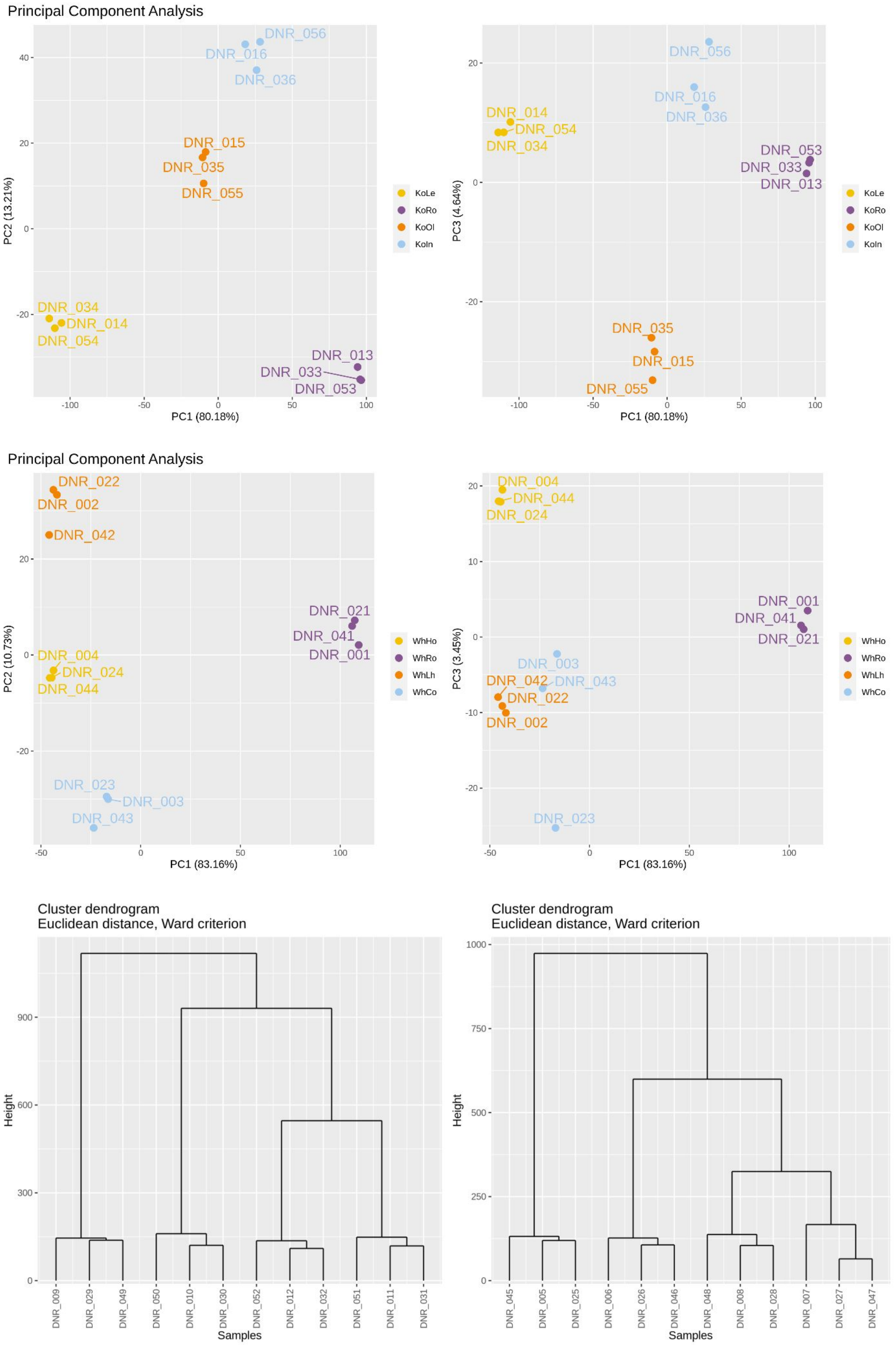
Principal Component Analysis
PC1 (80.18%)
PC2 (13.21%)
PC3 (4.64%)
KoLe
KoRo
KoOl
KoIn
DNR_056
DNR_016
DNR_036
DNR_015
DNR_035
DNR_055
DNR_034
DNR_014
DNR_054
DNR_013
DNR_033
DNR_053
Principal Component Analysis
PC1 (83.16%)
PC2 (10.73%)
PC3 (3.45%)
WhHo
WhRo
WhLh
WhCo
DNR_022
DNR_002
DNR_042
DNR_021
DNR_041
DNR_001
DNR_004
DNR_024
DNR_044
DNR_023
DNR_003
DNR_043
Cluster dendrogram
Euclidean distance, Ward criterion
Height
Samples
DNR_009
DNR_029
DNR_049
DNR_050
DNR_010
DNR_030
DNR_052
DNR_012
DNR_032
DNR_051
DNR_011
DNR_031
Cluster dendrogram
Euclidean distance, Ward criterion
Height
Samples
DNR_045
DNR_005
DNR_025
DNR_006
DNR_026
DNR_046
DNR_048
DNR_008
DNR_028
DNR_007
DNR_027
DNR_047

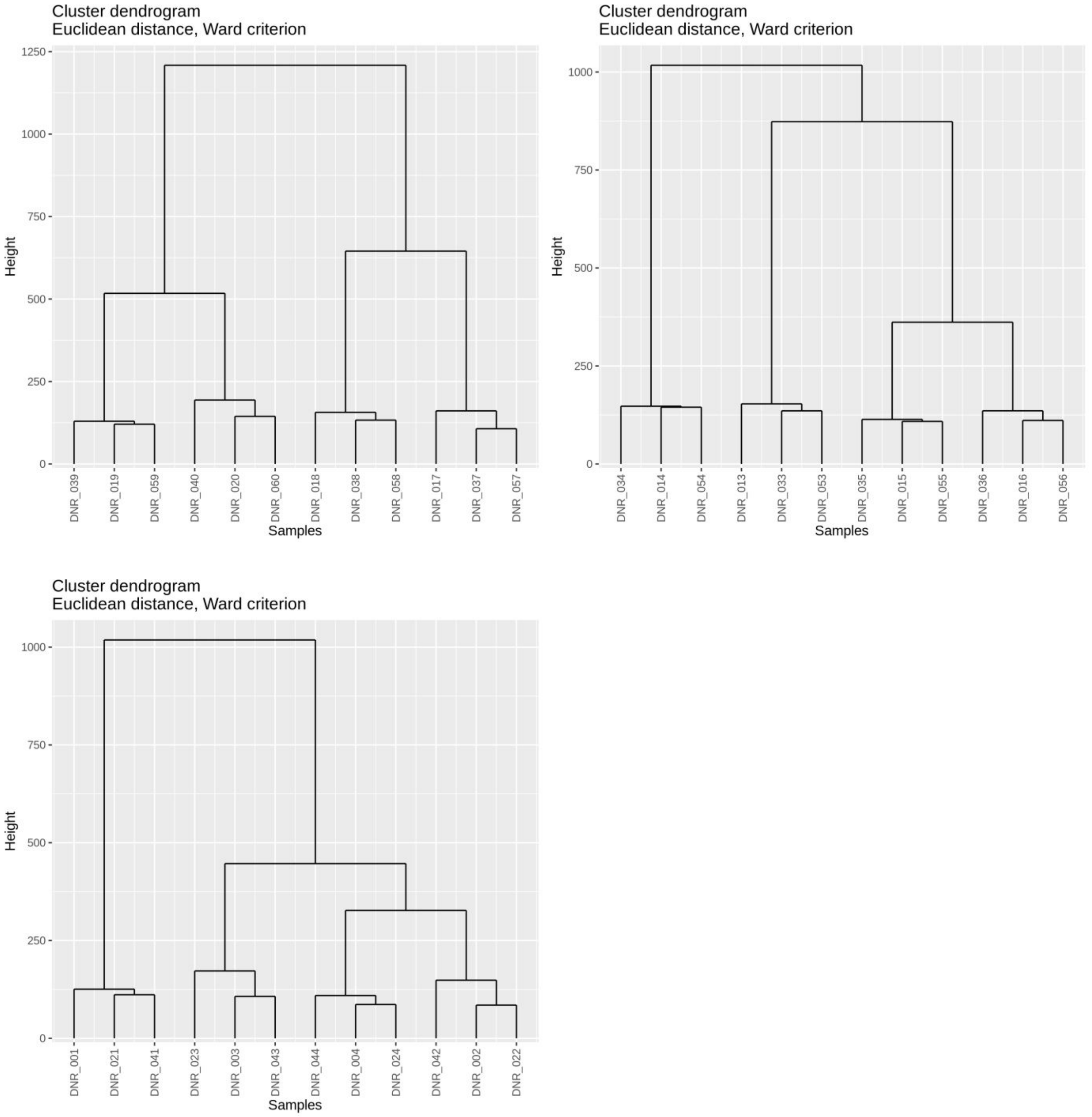

Fig. S5 PCA plots and cluster dendrogram based on mRNA-Seq data. In PCA plots, morphotypes from the top to bottom are: broccoli, cauliflower, kale, kohlrabi and white cabbage. Sample IDs in cluster dendrogram correspond to those used in PCA plots. Abbreviations in the PCA legends: BrLe: Broccoli Leaf, BrRo: Broccoli Root, BrIs: Broccoli infl_stem, BrFl: Broccoli Floret, CaLe: Cauliflower Leaf, CaRo: Cauliflower Root, CaIs: Cauliflower Infl_stem, CaFl: Cauliflower Floret, KaLm: Kale Leaf_mature, KaLy: Kale Leaf_young, KaSt: Kale Stem, KaRo: sssKale Root, KoLe: Kohlrabi Leaf, KoOl: Kohlrabi Outer_layer, KoIn: Kohlrabi Inner_part, KoRo: Kohlrabi Root, WhCo: White Core, WhLh: White Leaf_around_head, WhHo: White Head_in_outer, WhRo: White Root.

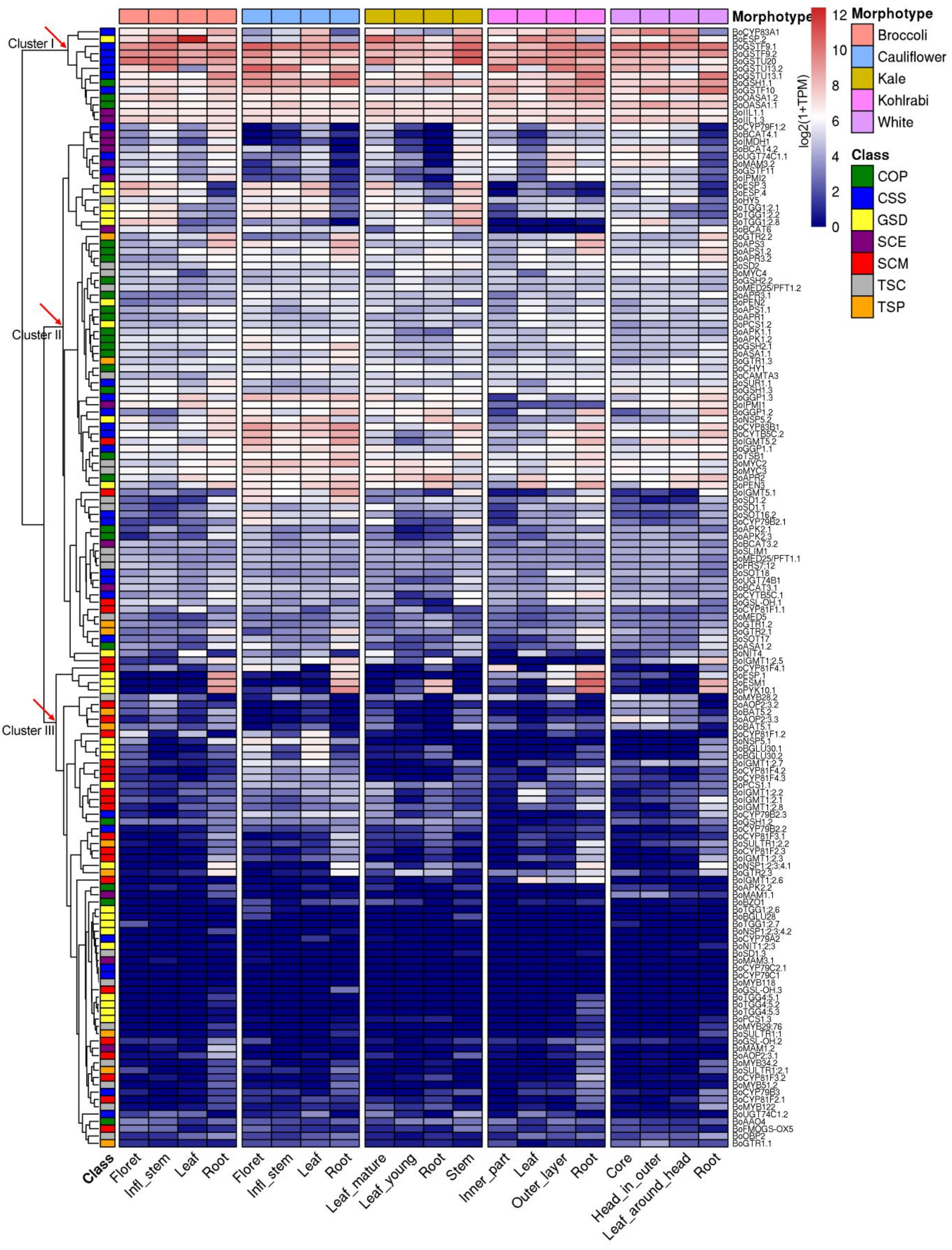

Fig. S6 Expression profiles for GSL related genes in four tissues of five *B. oleracea* morphotypes. Heatmaps were constructed using log2 transformed TPM values. Blue and red colors are used to represent low to high expression levels, respectively. Red arrows indicate nodes for the three clusters. Genes are classified based on their involvement in different processes/phases (The abbreviations: COP: Cosubstrate Pathways, CSS: Core Structure

Synthesis, GSD: GSL Degradation, SCE: Side-Chain Elongation, SCM: Side-Chain Modification, TSC: Transcriptional Components, TSP: Transporters).

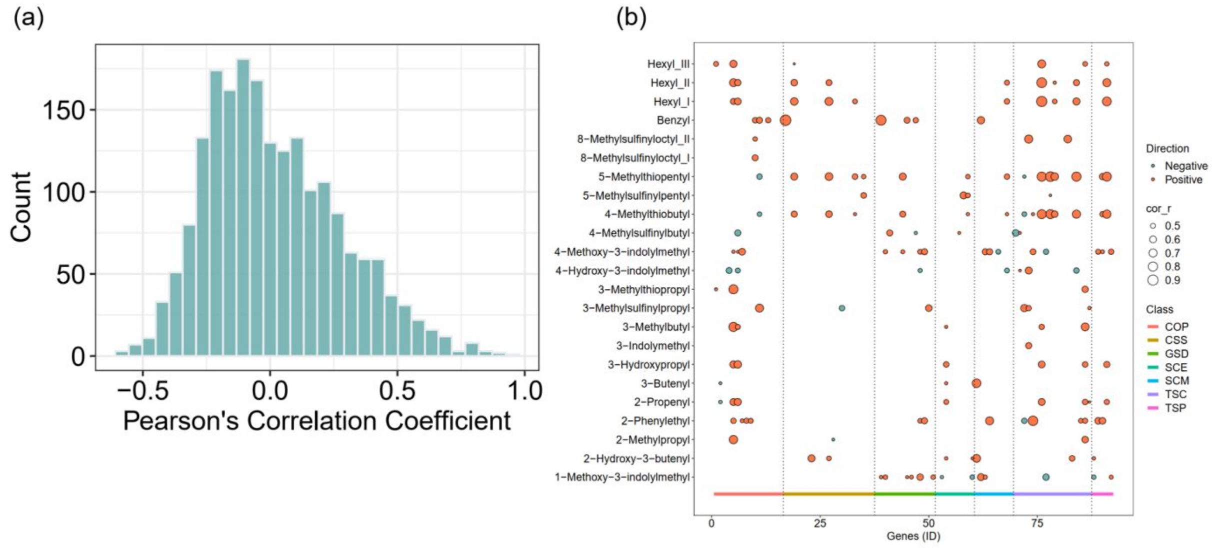

Fig. S7 Pearson's correlation analysis between GSLs and their related genes. Gene expression profiles (TPM values) are pooled for different copies of paralogous genes in *B. oleracea*. (a) Distribution of Pearson's correlation coefficient. (b) Significantly ($P < 0.05$) correlated GSLs and genes. Genes are classified based on their involvement in different processes/phases as shown in Fig. 2. (The abbreviations: COP: Cosubstrate Pathways, CSS: Core Structure Synthesis, GSD: GSL Degradation, SCE: Side-Chain Elongation, SCM: Side-Chain Modification, TSC: Transcriptional Components, TSP: Transporters).

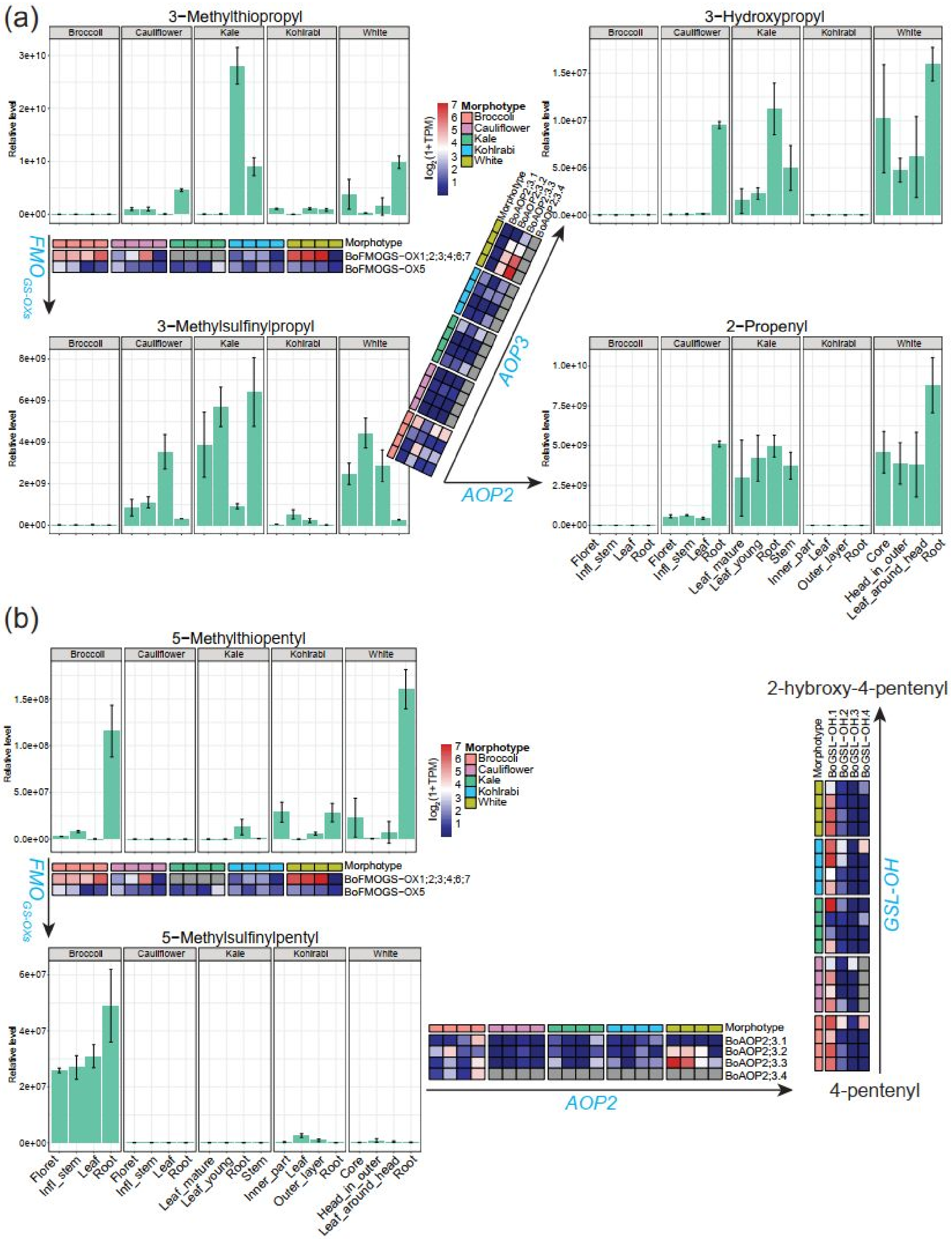

Fig. S8 C3 (a) and C5 (b) aliphatic GSL profiles and expression levels of related genes in different tissues and morphotypes. The bar charts show relative quantity of individual GSLs in respective tissues and morphotypes. Error bars indicate standard deviation (n = 3). Heatmaps show gene expression levels. Blue and red colors are used to represent low to high expression levels, respectively. Gray color denotes that the gene is not identified in the corresponding

morphotype. In all the bar charts and heatmaps, samples are displayed in the same order. Note: *BoAOP2;3.3* is *BoAOP2*.

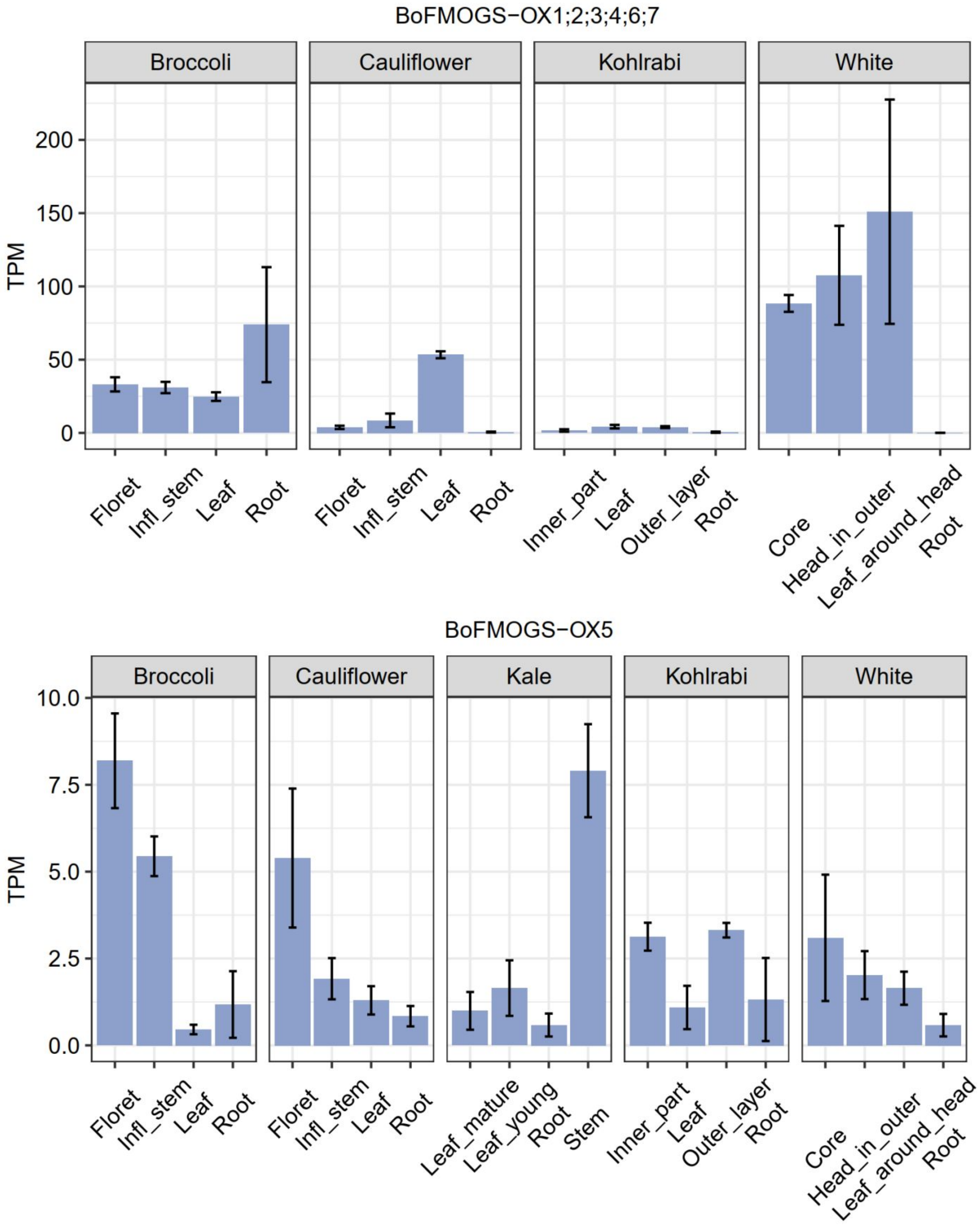

Fig. S9 Gene expression analysis of *FMO*$_{GS\text{-}OX}$ paralogues in four tissues in five *B. oleracea* morphotypes. The expression level was estimated using TPM values based on mRNA-Seq data. Error bars indicate standard deviation (n = 3).

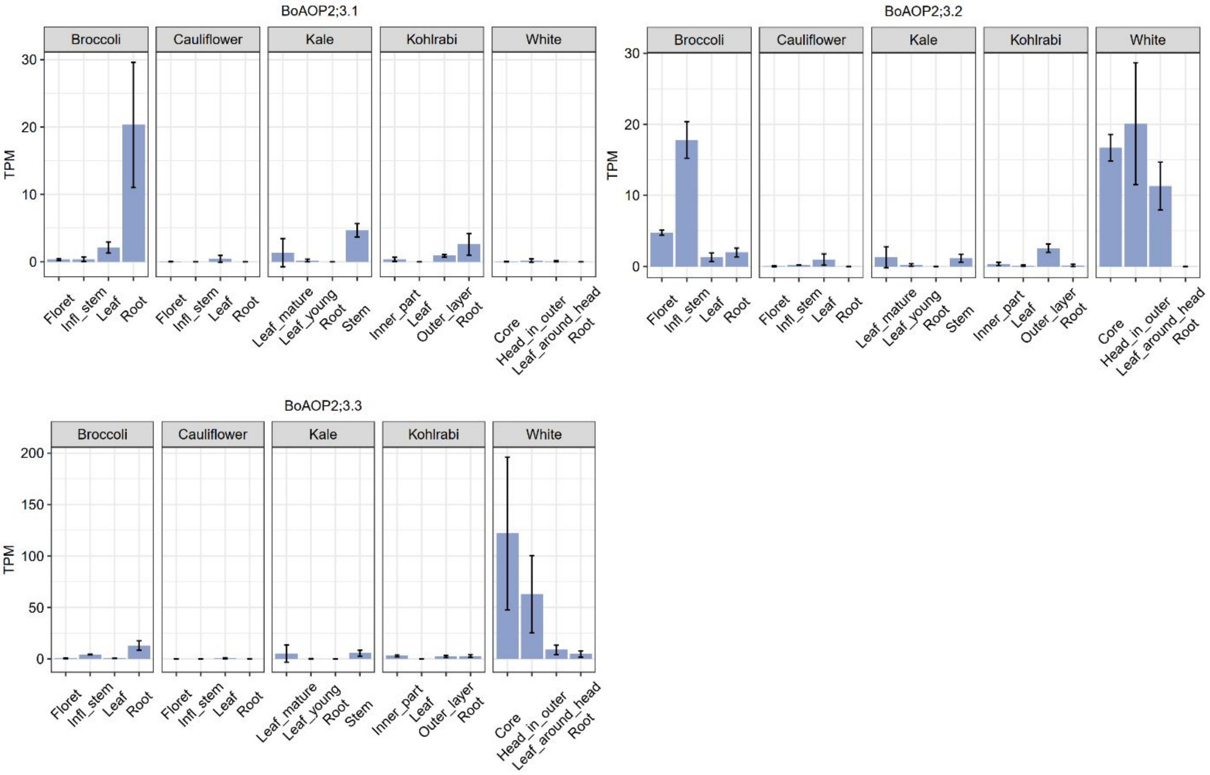

Fig. S10 Gene expression analysis of *AOP* paralogues in four tissues in five *B. oleracea* morphotypes. The expression level was estimated using TPM values based on mRNA-Seq data. Error bars indicate standard deviation (n = 3). Note: *BoAOP2;3.3* is *BoAOP2*.

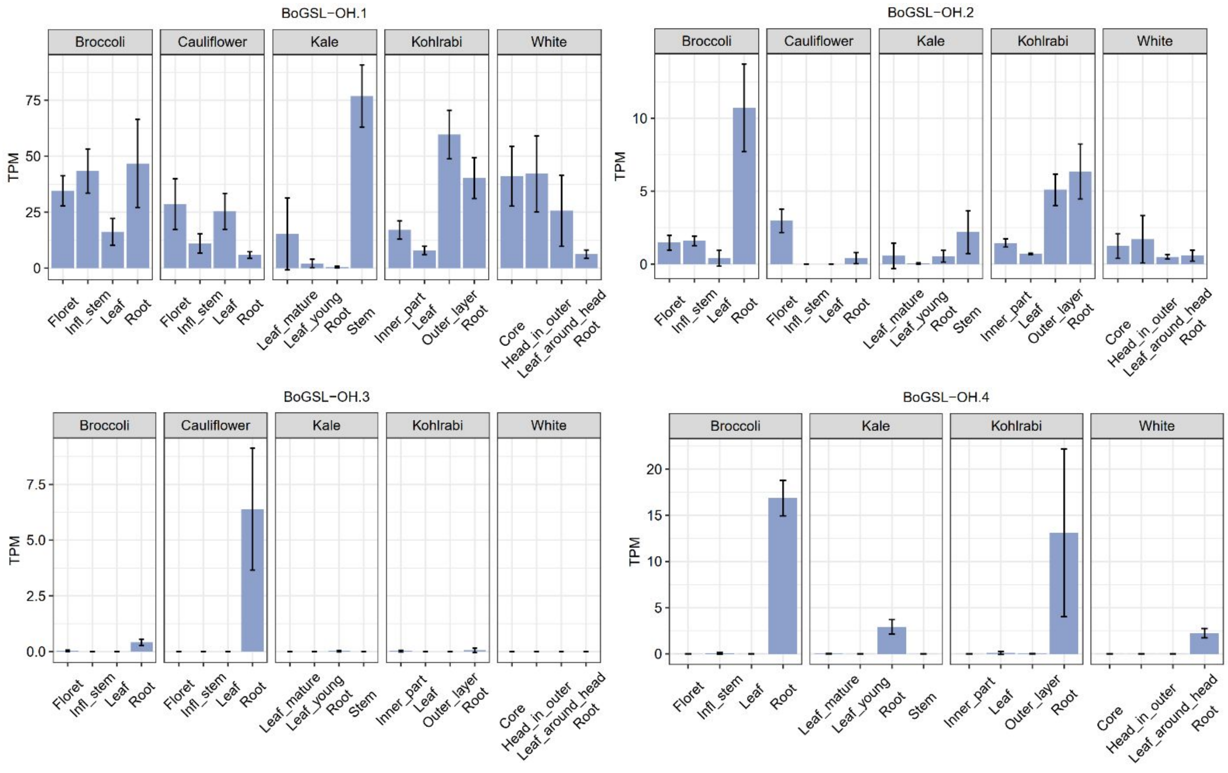

Fig. S11 Gene expression analysis of *GSL-OH* paralogues in four tissues in five *B. oleracea* morphotypes. The expression level was estimated using TPM values based on mRNA-Seq data. Error bars indicate standard deviation (n = 3).

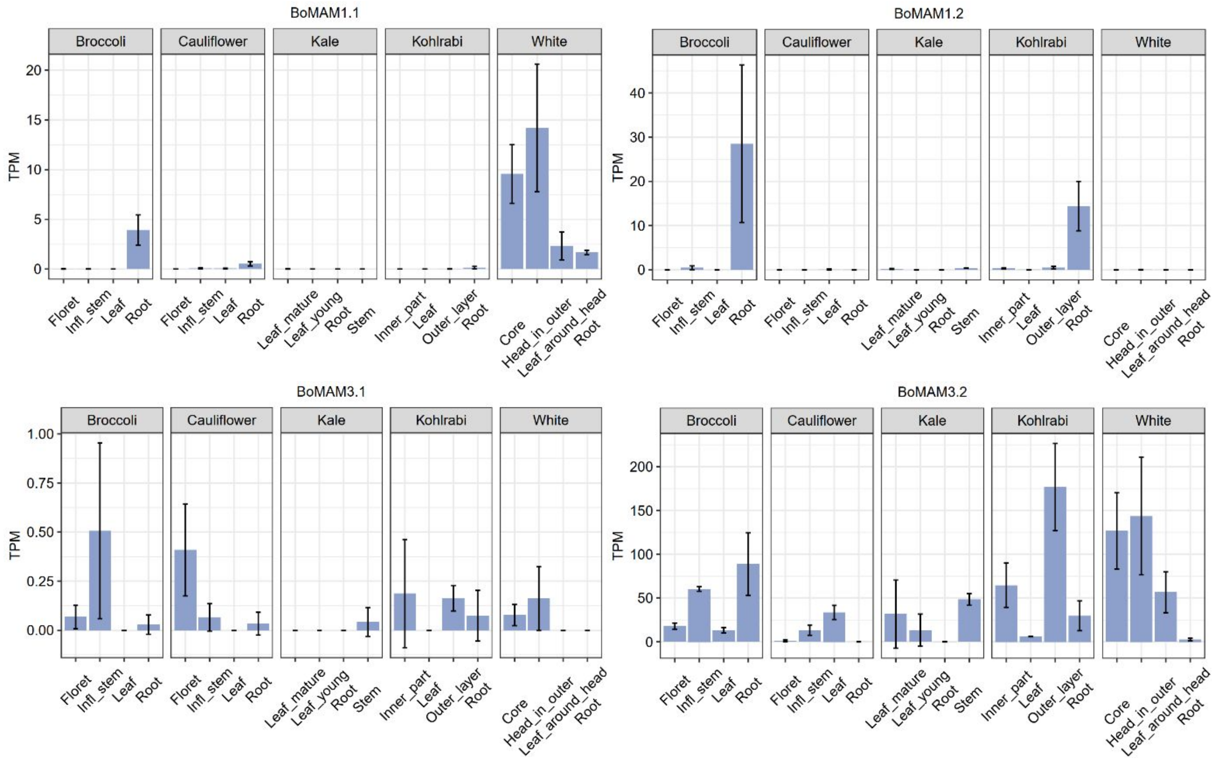

Fig. S12 Gene expression analysis of *MAM* paralogues in four tissues in five *B. oleracea* morphotypes. The expression level was estimated using TPM values based on mRNA-Seq data. Error bars indicate standard deviation (n = 3).

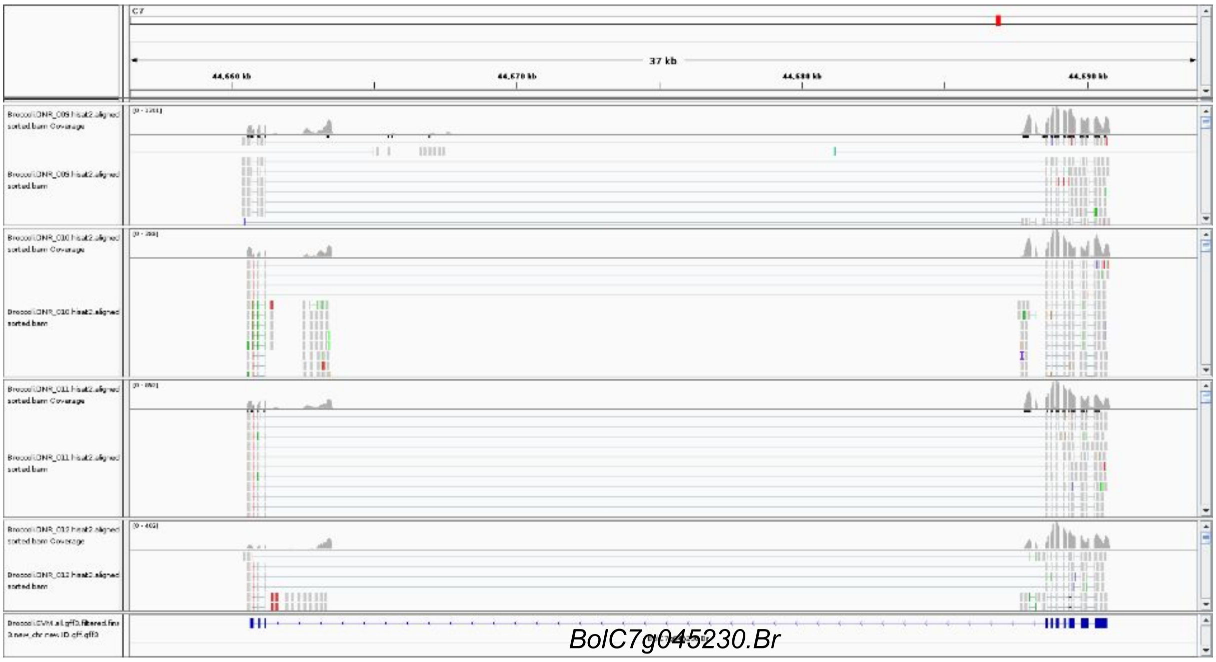
C7
37 kb
44,660 kb
44,670 kb
44,680 kb
44,690 kb
BolC7g045230.Br

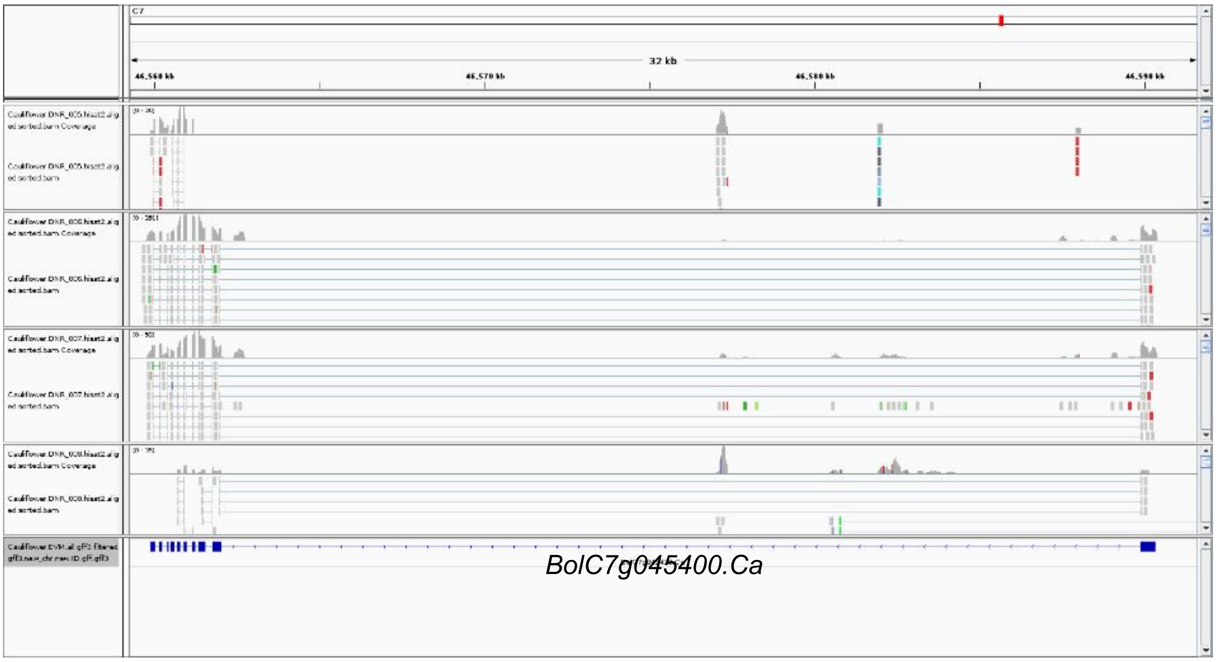
C7
32 kb
46,560 kb
46,570 kb
46,580 kb
46,590 kb
BolC7g045400.Ca

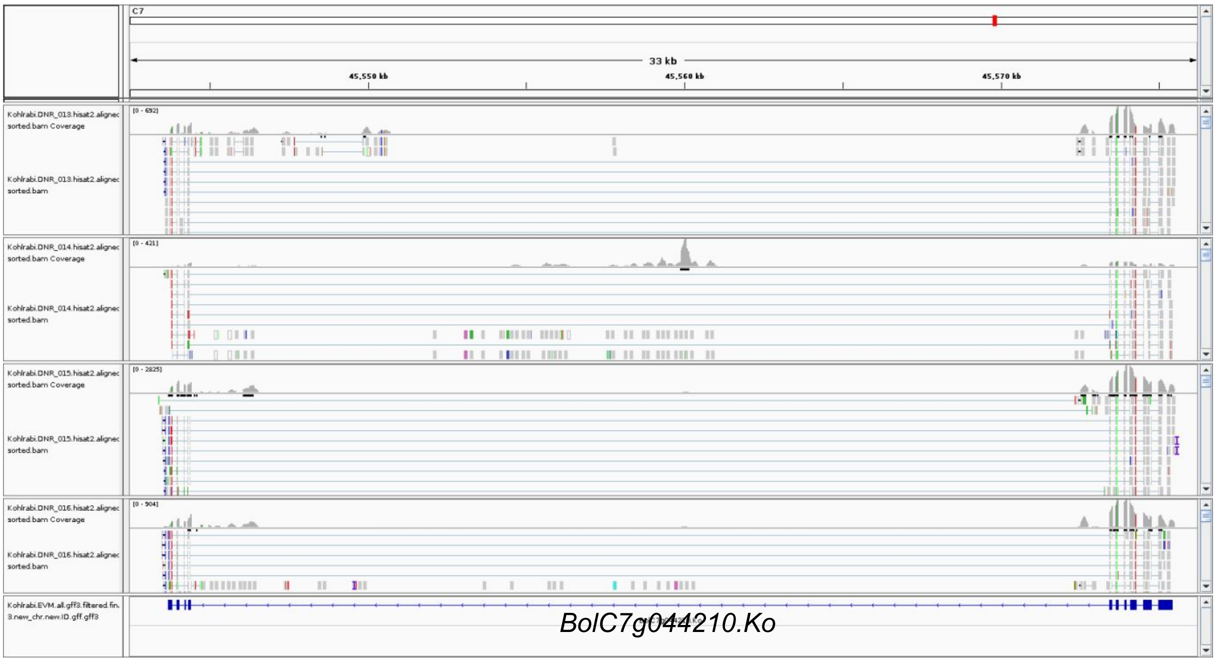
C7
33 kb
45,550 kb
45,560 kb
45,570 kb
Kohlrabi.DNR_013.hisat2.aligned.sorted.bam Coverage
Kohlrabi.DNR_013.hisat2.aligned.sorted.bam
Kohlrabi.DNR_014.hisat2.aligned.sorted.bam Coverage
Kohlrabi.DNR_014.hisat2.aligned.sorted.bam
Kohlrabi.DNR_015.hisat2.aligned.sorted.bam Coverage
Kohlrabi.DNR_015.hisat2.aligned.sorted.bam
Kohlrabi.DNR_016.hisat2.aligned.sorted.bam Coverage
Kohlrabi.DNR_016.hisat2.aligned.sorted.bam
BolC7g044210.Ko

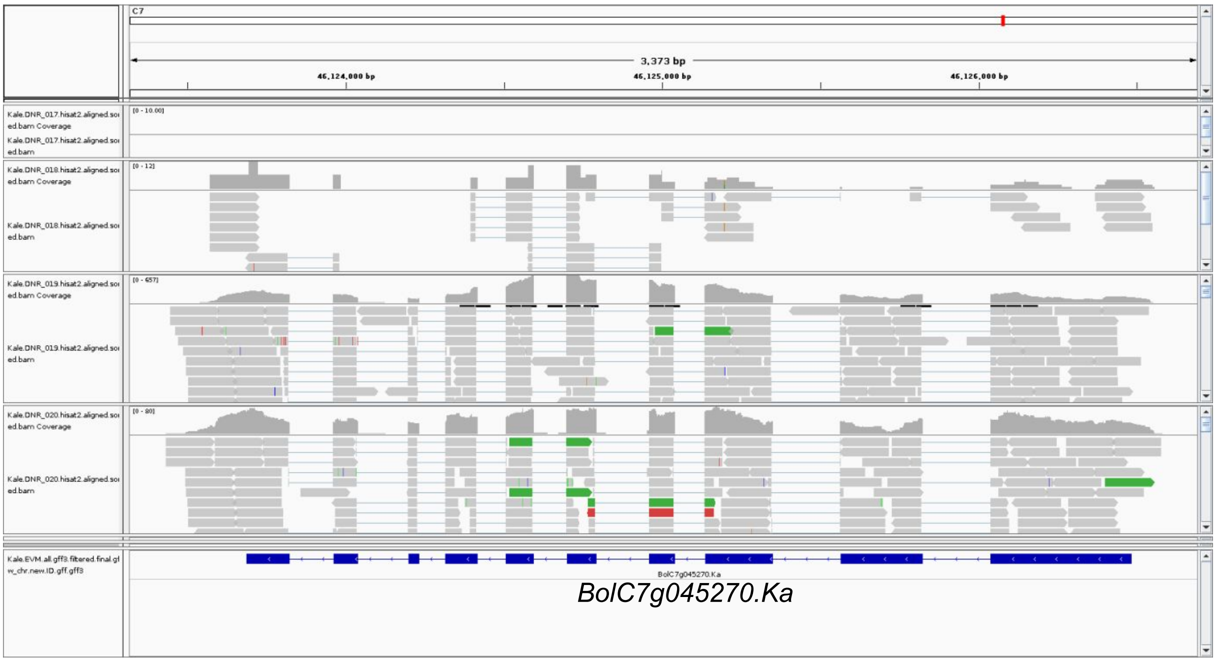
C7
3,373 bp
46,124,000 bp
46,125,000 bp
46,126,000 bp
Kale.DNR_017.hisat2.aligned.sorted.bam Coverage
Kale.DNR_017.hisat2.aligned.sorted.bam
Kale.DNR_018.hisat2.aligned.sorted.bam Coverage
Kale.DNR_018.hisat2.aligned.sorted.bam
Kale.DNR_019.hisat2.aligned.sorted.bam Coverage
Kale.DNR_019.hisat2.aligned.sorted.bam
Kale.DNR_020.hisat2.aligned.sorted.bam Coverage
Kale.DNR_020.hisat2.aligned.sorted.bam
BolC7g045270.Ka

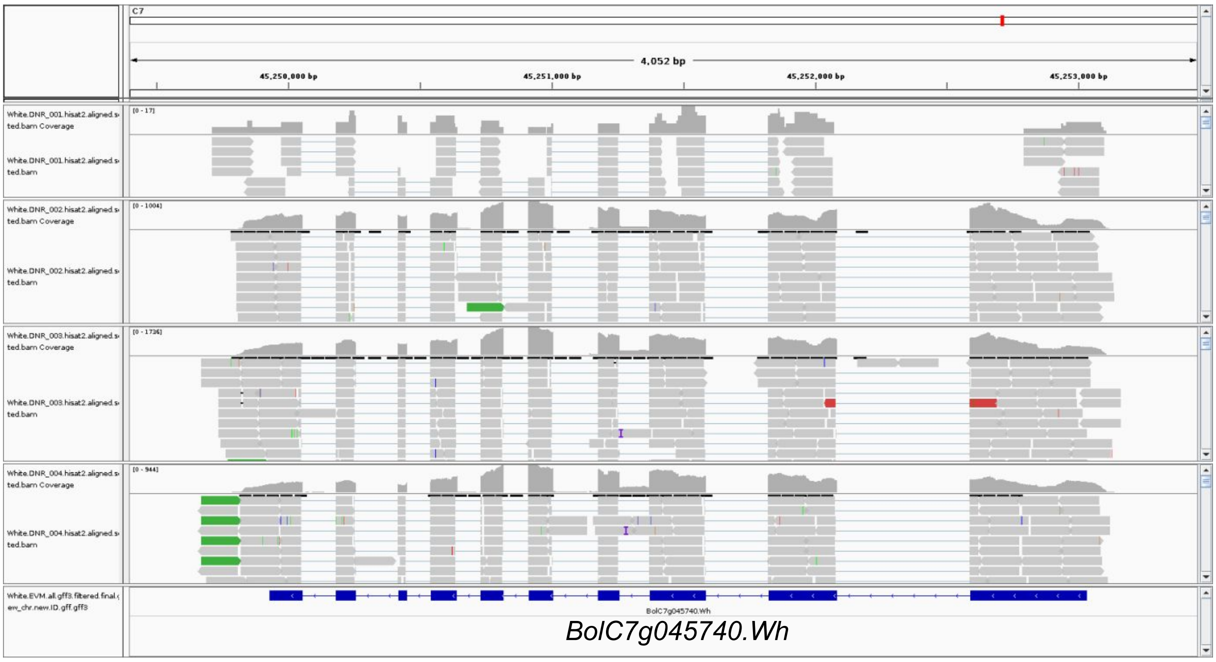

Fig. S13 IGV snapshots showing mRNA-Seq alignments in *BoMAM3.2* genes from five *B. oleracea* morphotypes (Top to bottom: broccoli, cauliflower, kohlrabi, kale and white cabbage). In each snapshot, four tracks from top to bottom represent alignments in four different tissues.

Table S1 Signature GSLs for the five *B. oleracea* morphotypes (Student-Newman-Keuls test with α=0.05). (Data shown in excel file)

Table S2 GSL related genes identified in the five *B. oleracea* genomes. (Data shown in excel file)

Table S3 Position of GSL related genes identified in the five *B. oleracea* genomes. (Data shown in excel file)

Table S4 Summary of RNA-Seq data and statistics for read mapping. (Data shown in excel file)

Table S5 List of significantly correlated GSLs and related genes. (Data shown in excel file)

Table S6 The number of GSLs/Genes that are significantly correlated with the given Gene/GSL. (Data shown in excel file)

Table S7 TE annotations in the long intron of *MAM3* gene in three *B. oleracea* genomes. (Data shown in excel file)

Table S8 Gene expression (TPM values) of GSL related genes in sampled tissues. (Data shown in excel file)

Table S9 Relative quantities of GSLs in different *B. oleracea* morphotypes and tissues. (Data shown in excel file)
